# Combinations of PARP Inhibitors with Temozolomide Drive PARP1 Trapping and Apoptosis in Ewing’s Sarcoma

**DOI:** 10.1371/journal.pone.0140988

**Published:** 2015-10-27

**Authors:** Sonja J. Gill, Jon Travers, Irina Pshenichnaya, Fiona A. Kogera, Syd Barthorpe, Tatiana Mironenko, Laura Richardson, Cyril H. Benes, Michael R. Stratton, Ultan McDermott, Stephen P. Jackson, Mathew J. Garnett

**Affiliations:** 1 Wellcome Trust Sanger Institute, Hinxton, United Kingdom; 2 The Wellcome Trust/Cancer Research UK Gurdon Institute and Department of Biochemistry, University of Cambridge, Cambridge, United Kingdom; 3 Massachusetts General Hospital Cancer Center, Harvard Medical School, Charlestown, Massachusetts, United States of America; University of South Alabama Mitchell Cancer Institute, UNITED STATES

## Abstract

Ewing’s sarcoma is a malignant pediatric bone tumor with a poor prognosis for patients with metastatic or recurrent disease. Ewing’s sarcoma cells are acutely hypersensitive to poly (ADP-ribose) polymerase (PARP) inhibition and this is being evaluated in clinical trials, although the mechanism of hypersensitivity has not been directly addressed. PARP inhibitors have efficacy in tumors with *BRCA1/2* mutations, which confer deficiency in DNA double-strand break (DSB) repair by homologous recombination (HR). This drives dependence on PARP1/2 due to their function in DNA single-strand break (SSB) repair. PARP inhibitors are also cytotoxic through inhibiting PARP1/2 auto-PARylation, blocking PARP1/2 release from substrate DNA. Here, we show that PARP inhibitor sensitivity in Ewing’s sarcoma cells is not through an apparent defect in DNA repair by HR, but through hypersensitivity to trapped PARP1-DNA complexes. This drives accumulation of DNA damage during replication, ultimately leading to apoptosis. We also show that the activity of PARP inhibitors is potentiated by temozolomide in Ewing’s sarcoma cells and is associated with enhanced trapping of PARP1-DNA complexes. Furthermore, through mining of large-scale drug sensitivity datasets, we identify a subset of glioma, neuroblastoma and melanoma cell lines as hypersensitive to the combination of temozolomide and PARP inhibition, potentially identifying new avenues for therapeutic intervention. These data provide insights into the anti-cancer activity of PARP inhibitors with implications for the design of treatment for Ewing’s sarcoma patients with PARP inhibitors.

## Introduction

Ewing’s sarcoma is a malignant bone tumour in which 85% of patients harbour a gene translocation involving the Ewing’s sarcoma breakpoint region 1 (*EWS*) gene fused to the Friend leukaemia virus integration site 1 (*FLI1*) gene: EWS-FLI1 t(11;22) [1, 2]. The translocation encompasses the N-terminal transcriptional activation domain of *EWS* and the C-terminal DNA binding domain of *FLI1*, which drives cellular transformation [1]. First-line treatment for Ewing’s sarcoma involves multidrug chemotherapy, radiotherapy, and/or surgical excision of the primary tumor, and is associated with high morbidity [3]. Moreover, 25% of patients present with metastatic disease and many relapse [4]. Prognosis is poor for these patients, with 5-year overall survival rates of 30% for patients with late recurrence, and 7% for patients who experience early recurrence [5, 6]. There is therefore a need for more targeted regimes with reduced treatment associated morbidity and long-term survival benefit of patients with Ewing’s sarcoma.

We previously reported a large-scale unbiased drug sensitivity screen in an extensive cancer cell line panel, and identified hypersensitivity of Ewing’s sarcoma cells (EWSCs) to distinct PARP inhibitor (PARPi) chemotypes [7]. Poly (ADP-ribose) polymerases (PARPs) comprise a group of ADP-ribosyl transferase enzymes, which transfer ADP-ribose from NAD^+^ onto their target proteins (PARylation), thereby regulating a wide array of cellular processes [8]. PARP1 and the related protein PARP2 are involved in repairing DNA single-strand breaks (SSBs). SSBs drive PARP1/2 (hereafter referred to as PARP) binding to DNA, catalysing a series of PARylation events that promote DNA repair processes [8]. Through its involvement in SSB repair, PARP has been exploited therapeutically. Olaparib, a potent PARPi, exhibits synthetic lethality in cells with *BRCA1/2* mutations, which confer deficiency in DNA double-strand break (DSB) repair mediated by homologous recombination (HR) [9, 10]. These cells have a high dependency on PARP1 and its role in SSB repair, and consequently they are hypersensitive to PARP inhibition. Olaparib has anti-tumour activity in *BRCA*-mutant breast, ovary and prostate cancers [9, 11–14]. Additional genetic modulators of PARPi sensitivity have been identified, such as mutations in the genes encoding ATM, ATR or PTEN, and elevated PARP1 expression is emerging as a measure of PARPi sensitivity [15–18].

Another mechanism of cytotoxicity has also been described for PARPi. By catalytically inhibiting PARP, PARPi also block auto-PARylation by PARP, required for its dissociation from DNA [19–21]. Thus, PARP inhibition can lead to the formation of cytotoxic trapped PARP-DNA complexes and the accumulation of DSBs. The ability of PARPi to trap PARP differs among PARPi, and is not solely linked to their ability to catalytically inhibit PARP [22, 23].

Following the observation that the *EWS-FLI1* genotype may serve as a biomarker for PARPi sensitivity, a clinical trial was initiated testing single-agent olaparib in Ewing’s sarcoma patients with recurrent disease, but clinical response endpoints were not met [24–27]. More recently, PARPi in combination with the DNA alkylating agent temozolomide has been shown to have potent anti-tumour activity in Ewing’s sarcoma xenograft and orthotopic models [24, 28, 29], and multiple clinical trials are currently evaluating the combination of PARPi together with temozolomide.

In order to inform on opportunities for implementing PARPi in the treatment of Ewing’s sarcoma, we investigated the underlying mechanism of PARPi hypersensitivity in EWSCs. Notably, the mechanism of PARPi sensitivity in EWSCs has hitherto not been directly evaluated despite the potent activity of PARPi *in vitro* and *in vivo*. Our study provides evidence that PARPi sensitivity in Ewing’s sarcoma is not due to an apparent defect in HR-mediated DNA repair, and instead is associated with acute sensitivity to trapped PARP-DNA complexes. Furthermore, we identify a subset of glioma, neuroblastoma and melanoma cells that are particularly sensitive to a combination of temozolomide and PARPi, thereby potentially extending the clinical use of PARPi.

## Materials and Methods

### Cell lines and compounds

See supplementary methods (S1 Text) for a complete list of cell lines and culture conditions. Compounds were purchased from commercial vendors and stored as aliquots at -80°C subjected to a maximum of five freeze-thaw cycles.

### Drug sensitivity data

An unpaired two-sample t-test was performed on the natural log of IC_50_s of *EWS-FLI1*-mutant and wild-type cells with 95% confidence intervals using GraphPad Prism. We have included a table of cell line drug sensitivity data for the inhibitors used in this study (S1 Data). Genomic characterization of cell lines and generation of drug sensitivity data was performed as previously described [7].

### Cellular assays

Long term cell growth assays were conducted as previously described [7]. OLAR5 cells were generated by serial drug exposure [30]. Cells were assayed and drug treated in 96-well plates [7]. Cell viability was measured after 72h using Cell Titer Blue (Promega) or sulphorhodamine (SRB) colorimetric assay (Sigma), and apoptosis after 48h using ApoOne (Promega) as per manufacturer’s instructions. IC_50_s and concentrations for 50% of maximal inhibition of cell proliferation (GI_50_) were determined using GraphPad Prism software.

For combination drug screening, cells were plated in 384-well plates and drugs added in a 5x5 4-fold drug dilution matrix for 72h using robotics. Cells were analyzed using Syto60 (Invitrogen) and quality control performed as previously described [7].

### Immunofluorescence

For immunofluorescent analysis on the Cellomics Arrayscan (Thermo Fisher Scientific), cells were plated and treated on 96-well plates, fixed and permeabilized with 4% paraformaldehyde/0.1% Triton-X-100/PBS and washed with PBS. Cells were blocked (2%BSA/PBS) and incubated with 0.4μg/ml anti-γH2AX antibody (05–636; Millipore). Cells were washed (0.1% Tween/H_2_O) and incubated with 4μg/ml Alexa 488-labelled secondary antibody and 4μg/ml Hoechst (Sigma-Aldrich). Cells were washed and overlaid with PBS. All images were captured at 40x magnification and analyzed using the spot detector bioapplication. Cells were gated as positive for γH2AX with 4–5 foci per nucleus.

For confocal microscopy, cells were split onto coverslips in 6-well plates, labeled with 5μM EdU for 15 minutes prior to drug treatment, fixed (4% paraformaldehyde/PBS) and permeabilized (0.2% Triton/PBS). EdU was detected by Click-IT (Life Technologies). Cells were washed with PBS and coverslips treated with DNaseI for 2h in a 37°C humidified container. Cells were washed and blocked (1% BSA/2% FCS/PBS) and stained with 0.2μg/ml anti-RAD51 antibody (H-92; Santa Cruz Biotechnologies) and 0.2μg/ml anti-γH2AX antibody (05–636; Millipore) overnight. Cells were washed with PBS, stained with 4μg/ml Alexa 555-labelled secondary antibody then washed and stained with 1μg/ml DAPI (Sigma) on a rocking platform. Each coverslip was rinsed in distilled water, blotted dry and mounted onto a slide by inverting into a drop of vectashield (Vector Laboratories) and analysed on an Olympus confocal microscope and a Deltavision fluorescence microscope with 40x and 100x objectives respectively.

### Western immunoblotting

See supplementary methods (S1 Text) for details of cell lysate preparation, quantification and antibodies used. Cellular sub-fractionation assays were performed using a kit as per manufacturer’s instructions (Thermo Fisher Scientific).

### siRNA depletions

Cells were either mock-transfected or transfected with a scrambled negative control (Ambion), siPARP1_1 (CCAUUGAGCACUUCAUGAA; Sigma), siPARP1_2 (GAUAGAGCGUGAAGGCGAA; Sigma), siCtIP (GCUAAAACAGGAACGAAU) or siBRCA1 (GGAACCUGUCUCCACAAG) in a reverse format using RNAiMax (Life Technologies). To determine depletion efficiency, cells were transfected in 6-well plates and lysed at the time of drug treatment (18h post-transfection). Otherwise cells were transfected in 96-well plates, drug treated 18h post-transfection and viability assays performed 96h post-drug treatment whereas cells were fixed for immunofluorescent analysis 8h post-drug treatment. For assessment of olaparib sensitivity following BRCA1 and CtIP depletion, cells were drug treated 48h post-transfection and assayed 96h later.

## Results

### EWSCs are hypersensitive to PARP inhibition and S-phase DNA-damaging agents

**Fig 1:**
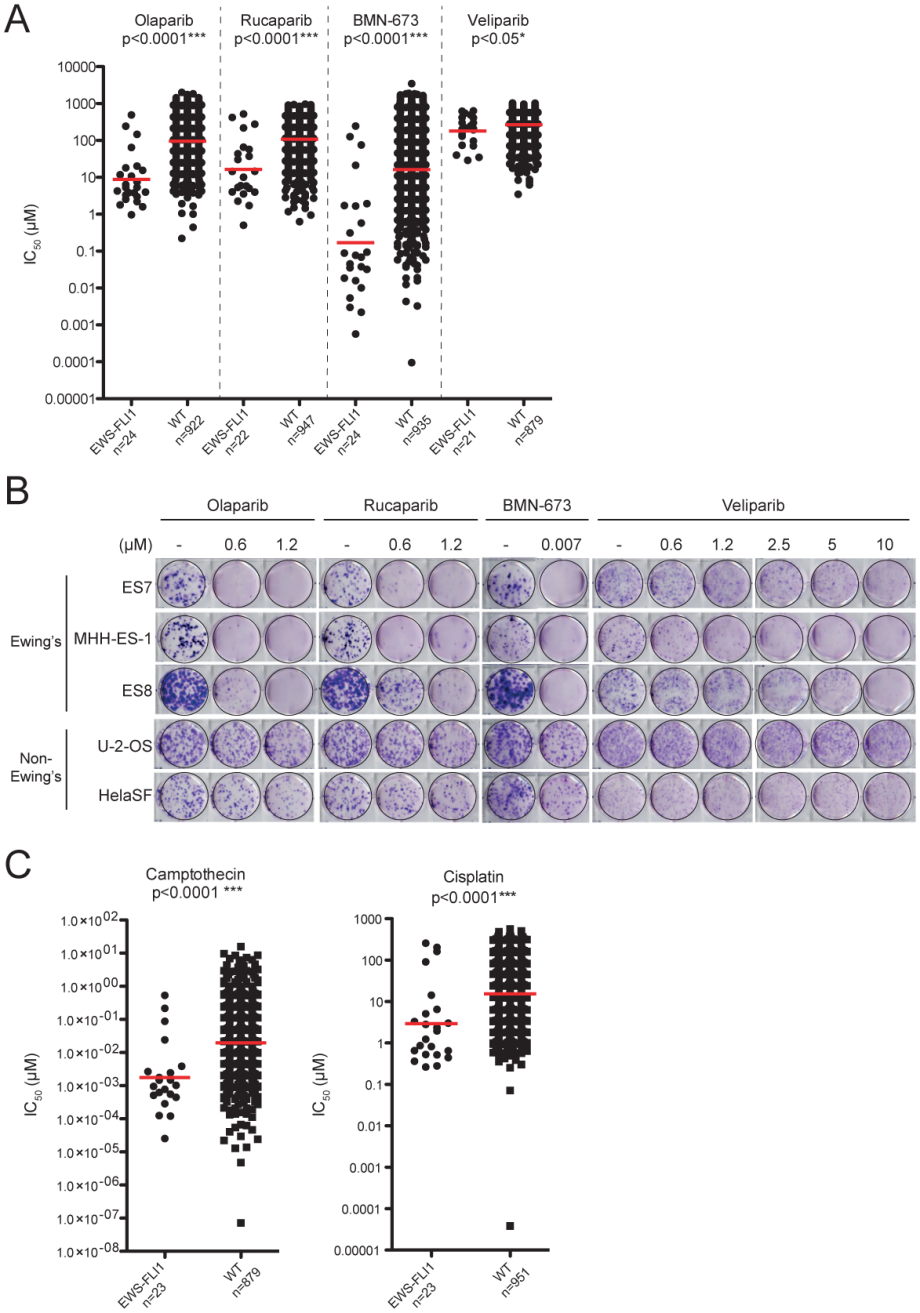
EWSCs are sensitive to PARP inhibition and S-phase DNA-damaging agents. **(A)** and **(C)** Scatter plots of IC_50_ (μM) values on a log scale comparing drug sensitivity of *EWS-FLI1*-positive and wild-type (WT) *EWS-FLI1*-negative cell lines to (A) four PARPi and (C) camptothecin and cisplatin. The sample size (n) is indicated and each circle represents the IC_50_ of one cell line. The red bar is the geometric mean and the drug name is depicted above each plot along with the significance of the association as determined by an unpaired two-sample t-test. **(B)** Long term viability assays in EWSCs were performed in the presence of vehicle (-) or increasing concentrations of four PARPi as indicated. Non-EWSC lines (U-2-OS and HeLaSF) are included for comparison. These data are representative of 3 independent experiments.

We previously performed a large-scale drug sensitivity screen in >400 cancer cell lines and identified a marked hypersensitivity of EWSCs to PARP inhibition [7]. This sensitivity was detected in a 72-hour assay and resulted in apoptosis. Here, we extend these results by screening >950 cancer cell lines against the PARPi olaparib (AZD-2281), rucaparib (AG-014699), veliparib (ABT-888) and BMN-673 [31–35]. To validate *EWS-FLI1* as a marker of sensitivity, we confirmed disruption of the *EWS* gene in all the EWSCs in our cell panel (S1A Fig). These studies confirmed a marked hypersensitivity of EWSCs to three of the four PARPi (BMN 673 > olaparib > rucaparib) (Fig 1A). This was validated in 10–14 day long term cell growth assays, and sensitivity was observed at concentrations as low as 7nM for BMN-673, and 600nM for olaparib and rucaparib (Fig 1B) [7]. In contrast, veliparib showed only marginal activity against EWSCs in our screen, and in long term growth assays we observed only partial sensitivity at 1.2–10μM (Fig 1A and 1B). In this regard, we note that, despite veliparib potently inhibiting PARP catalytic activity at concentrations >1 μM it has reduced trapping efficiency compared to other PARP inhibitors [22].

We found that EWSCs are also markedly hypersensitive to S-phase DNA-damaging agents including camptothecin analogs, bleomycin, cisplatin, gemcitabine and doxorubicin (Fig 1C and S1B Fig) [7]. However, sensitivity to inhibitors of other DNA-damage response (DDR) components including ATM, ATR, DNA-PK, CHK1 or CHK2 was not observed (data not shown). Thus, EWSCs are specifically hypersensitive to PARPi and S-phase DNA-damaging agents.

### Olaparib induces DNA DSBs despite functional DDR and HR in EWSCs

We sought to investigate the mechanism of sensitivity of EWSCs to PARP inhibitors, focusing on a representative cell line ES8 and the clinically approved drug Lynparza^TM^ (olaparib) [36]. We verified our results by using multiple different PARPi with additional EWSC lines (MHH-ES-1 and ES7). Whole-exome sequencing of EWSCs did not identify mutations in DNA repair genes as a possible reason for the observed sensitivity (sequencing data available on COSMIC) [37]. We examined levels of DDR proteins including ATM, ATR, 53BP1, CHK1, CHK2, MRE11, BRCA1 and BRCA2 by western immunoblotting, all of which were expressed in EWSCs (S2A and S2B Fig).

**Fig 2:**
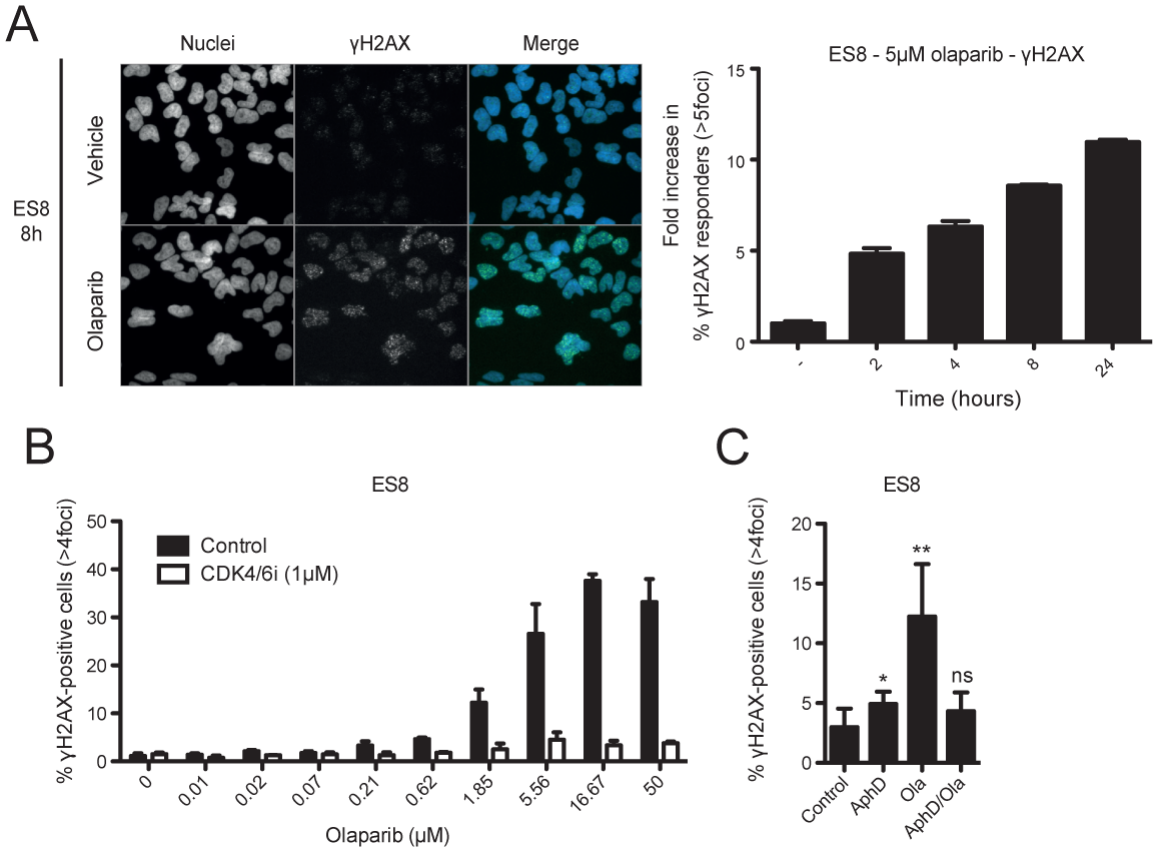
Olaparib induces DNA DSBs in S-phase of the cell cycle in EWSCs. **(A)** ES8 cells were treated with vehicle or olaparib and stained with Hoechst (nucleus; blue) and for γH2AX (DSBs; green). Images on the left are of the 8-hour time point. The graph measures fold increase in γH2AX responders at the time points indicated. Error bars represent the standard deviation of the mean of technical triplicates. **(B)** ES8 cells were treated with olaparib for 16 hours following a 6-hour pre-treatment with palbociclib (CDK4/6i) or vehicle and percentage of γH2AX responders determined. **(C)** ES8 cells were treated with vehicle, 5μM aphidicolin (AphD), 5μM olaparib (Ola) or a 30-minute pre-treatment with aphidicolin followed by olaparib for 8 hours and percentage of γH2AX responders determined. Asterisks indicate *student’s paired t-test P* value *P<0.05, **P<0.01, ns = not significant, relative to control. These data are representative of 3 independent experiments.

We then characterized the effect of olaparib on genome integrity. Serine-139 phosphorylated histone H2AX (γH2AX), a marker of DNA DSBs, was rapidly induced within 2 hours of olaparib treatment and steadily increased over 24 hours, and this response was dose-dependent (Fig 2A and 2B and S3A Fig). Induction of 53BP1 foci was also observed, suggestive of on-going DNA repair (S3B Fig). Notably, blocking entry into S-phase of the cell cycle with a CDK4/6 inhibitor (palbociclib), or inhibiting replication with aphidicolin, prevented accumulation of γH2AX following olaparib treatment, indicating that progression into S-phase and on-going replication are necessary for overt induction of DNA damage by olaparib in EWSCs (Fig 2B and 2C and S3A and S3C Fig).

**Fig 3:**
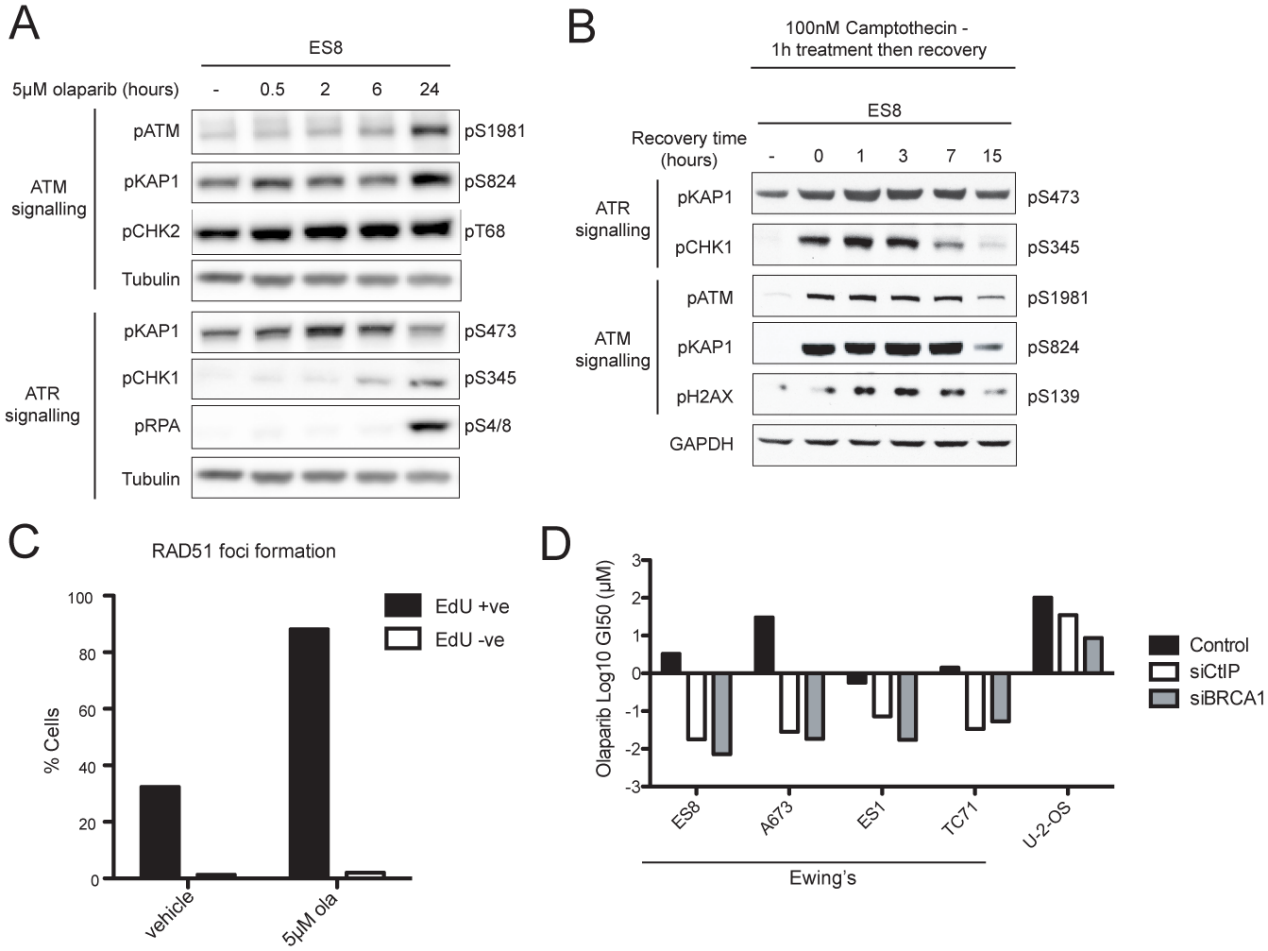
DNA DSB repair by HR is functional in EWSCs. **(A)** Western blot of ES8 cells treated with olaparib for the times indicated. Markers are grouped as part of ATM or ATR signaling. Tubulin served as a loading control. (**B)** Western blot of ES8 cells treated with camptothecin and harvested at various time points following drug washout. GAPDH served as a loading control. **(C)** Percentage of EdU-positive and EdU-negative ES8 cells with >5 nuclear RAD51 foci following 6-hour treatment with vehicle or olaparib (ola). **(D)** Olaparib log GI_50_ (μM) of cell lines mock-transfected or transfected with CtIP or BRCA1 siRNA as indicated.

We then examined whether the ATM and ATR pathways involved in signaling DNA damage were functional in EWSC. ATM is typically activated in response to DSBs, promotes DSB exonuclease processing, and activates an S-phase checkpoint [38]. ATR slows down S-phase progression and mitotic entry, to enable protection and restart of stalled replication forks. In ES8 cells, olaparib treatment induced autophosphorylation of ATM (Ser-1981), and phosphorylation of its downstream targets KAP1 (Ser-824) and CHK2 (Thr-68) (Fig 3A). We also observed phosphorylation of RPA (Ser-4/8), an early marker of DSB-resection and HR, as well as activation of CHK1 (phosphorylated Ser-345), both of which are DDR markers associated with ATR activation. KAP1 Ser-473 is phosphorylated by CHK1, and was also induced [39]. Similar results were observed in multiple EWSCs, and also in response to camptothecin, which induces DSBs by trapping topoisomerase I (Fig 3B and S4A Fig). Collectively, these data indicated that ATR and ATM signaling are functional in EWSCs.

A key step in HR is recruitment of RAD51 to sites of DNA damage, facilitating homology search and recombination, an event notably impaired in cancer cells that harbor deficiencies in HR [10]. Following olaparib treatment of EWSCs, we observed induction of RAD51 foci in S-phase cells, labeled by EdU-incorporation during drug treatment, suggesting that HR is functional to a late stage in such cells and further demonstrating that DSBs accumulate in actively replicating EWSCs (Fig 3C and S3D Fig). To further test the proficiency of HR in EWSCs, we depleted critical HR proteins, BRCA1 and CtIP (or RBBP8), by siRNA [40]. Notably, although EWSCs are hypersensitive to PARPi, four representative EWSCs were further sensitized to olaparib by depletion of BRCA1 or CtIP, revealing that these factors act in EWSCs to mitigate olaparib toxicity (Fig 3D). Thus, although we cannot completely exclude a defect in the DDR in EWSCs, our results demonstrate that HR is at least partially operational in EWSCs, and that ATM and ATR DDR pathways involved in detecting, signaling and responding to DNA damage are functional. Notably, further support for functional repair pathways in EWSCs comes from the exceptionally low burden of mutations and structural variation observed in the tumours of Ewing’s sarcoma patients compared to other malignancies [41].

### EWSCs are hypersensitive to PARP1 trapping

**Fig 4:**
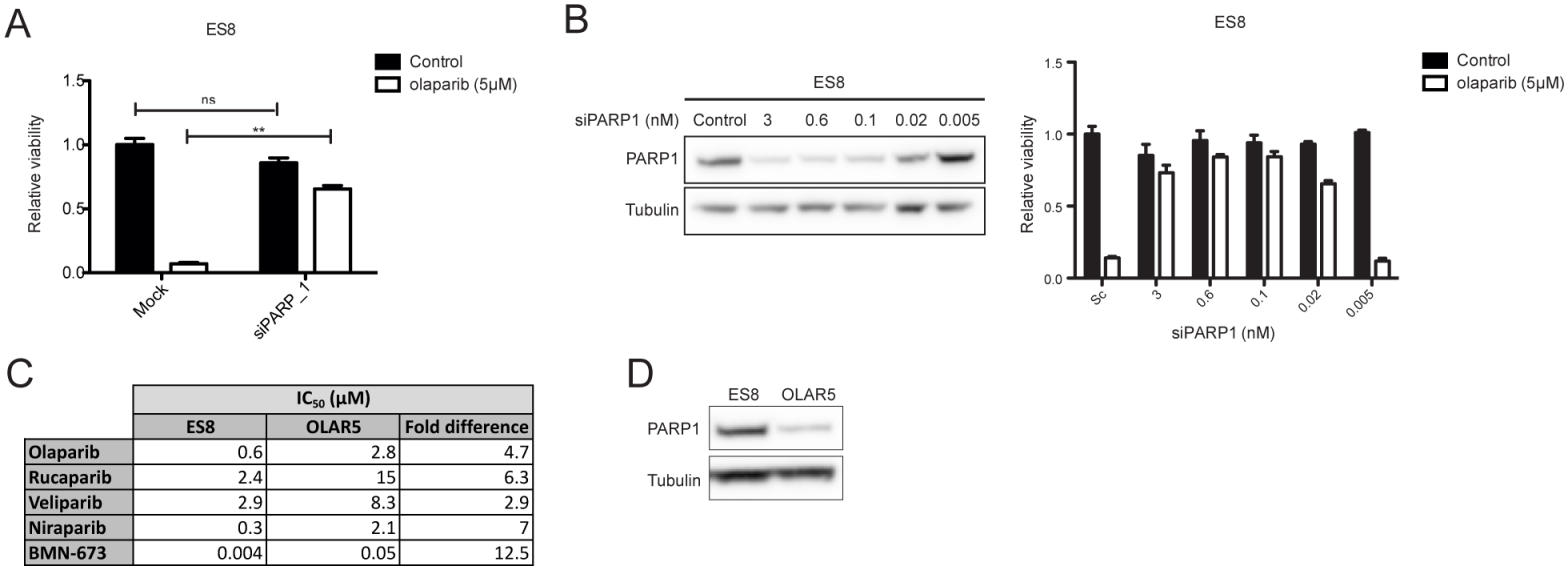
EWSCs are sensitive to PARP1 trapping. **(A)** Relative viability of mock-transfected and PARP1 siRNA-transfected ES8 cells treated with vehicle or olaparib. Asterisks indicate *student’s paired t-test P* value **P<0.01, ns = not significant. **(B)** PARP1 expression in cells transfected with a scrambled control or a titration of PARP1_1 siRNA and their relative viability following treatment with vehicle or olaparib. **(C)** IC50 values of parental ES8 and PARPi-resistant OLAR5 cells to five different PARPi and the fold difference between them. **(D)** Western blot of PARP1 expression in ES8 and OLAR5 cells. Viability values are the mean of technical triplicates and representative of 3 independent experiments.

The hypersensitivity of EWSCs to multiple PARPi and the absence of an apparent DDR defect suggested that PARP trapping underpins sensitivity. To test this, we depleted PARP1 with siRNA and measured the effect on viability in ES8 cells. PARP1 siRNA efficiently depleted PARP1 in ES8 cells but depletion alone had little effect on viability (Fig 4A black columns and S4B Fig). Notably, however, PARP1 depletion reversed the sensitivity of ES8 cells towards olaparib and also the structurally and chemically distinct PARPi rucaparib (Fig 4A white columns and S4C Fig), and by using a titration of PARP1 siRNA we observed that the extent of the reversal correlated with PARP1 expression levels (Fig 4B). Similar effects were observed in ES7 and MHH-ES-1 cells, and when using two different siRNA targeting PARP (S4B, S4D and S4E Fig). Moreover, we generated a PARPi-resistant clone of ES8 cells by serial olaparib exposure, named OLAR5, which had substantially enhanced resistance to multiple PARPi compared to parental cells (Fig 4C). Strikingly, we found that OLAR5 cells had strongly down-regulated PARP1 protein expression (Fig 4D), further suggesting that PARP1 protein is required for the toxicity of PARPi in EWSCs and consistent with sensitivity observed in prostate cancer and chicken DT40 cells where PARP trapping is operative [22].

PARP inhibition in combination with DNA alkylating agents has potent anti-tumour activity in Ewing’s sarcoma xenograft and orthotopic models [24, 29], and the use of PARP inhibitors (olaparib, niraparib and BMN-673) with temozolomide is currently being evaluated in clinical trials (NCT02044120, NCT01858168 and NCT02116777). Thus, we decided to investigate whether the underlying mechanism of sensitivity to this combination was also driven by hypersensitivity to PARP trapping and if so, whether PARP trapping was only enhanced by alkylating agents or also by other S-phase damaging agents with different modes-of-action.

**Fig 5:**
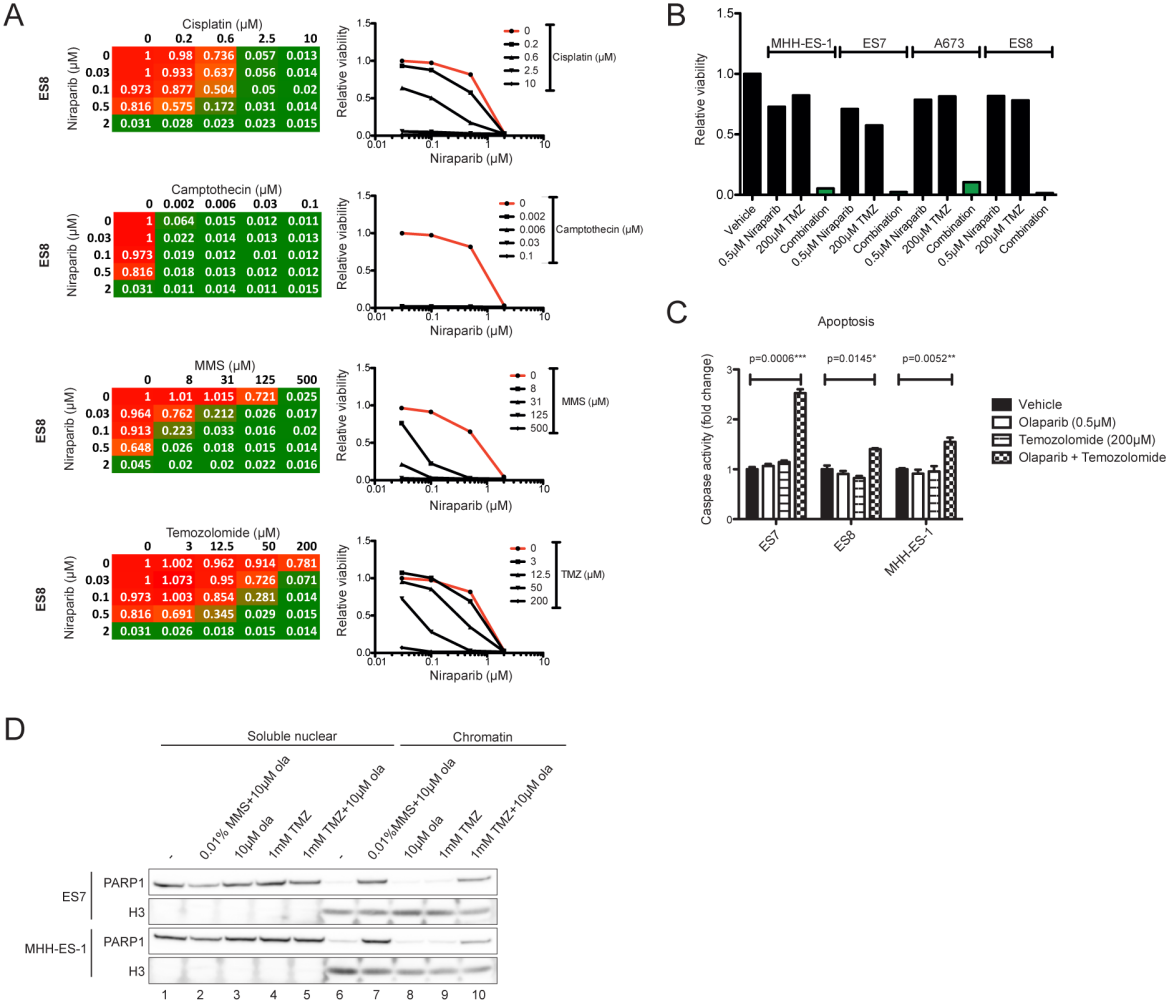
Temozolomide enhances olaparib-induced PARP1 trapping. **(A)** Heatmaps (left panel) of relative viability values of ES8 cells screened against a combination of niraparib and one of three chemotherapies or MMS. High viability values are in red and low viability values in green. Graphs (right panel) show the corresponding dose response curves measuring relative viability with a separate line plotted for each concentration of the combined drug. The dose response for niraparib alone is highlighted in red. Viability values are the mean of technical duplicates. **(B)** Relative viability of EWSCs treated with vehicle, niraparib (0.5uM) or temozolomide (TMZ) alone (200uM), or in combination. The combination effect is highlighted in green. **(C)** Fold induction of caspase 3/7 activation in EWSCs following treatment with vehicle, olaparib or temozolomide alone or in combination for a total of 48 hours. A *student’s paired t-test* was performed and significance values are indicated. **(D)** Cellular sub-fractionation assay following treatment of EWSCs with vehicle (-), MMS in combination with olaparib (ola), or olaparib and temozolomide (TMZ) alone or in combination for 4 hours.

The DNA alkylating agent methyl methane sulfonate (MMS) drives accumulation of methyl-DNA adducts, repair of which is promoted by PARP DNA-binding and enhances PARP trapping [22, 23, 42]. Thus, to evaluate whether S-phase DNA damaging agents enhance PARP1 trapping in EWSCs, we performed a screen of multiple PARPi (rucaparib, niraparib and BMN-673) in combination with three clinically used S-phase damaging agents with distinct modes-of-action (cisplatin, temozolomide and camptothecin), and included MMS as a positive control. Niraparib was selected because it was the only PARPi in clinical trials with temozolomide at the time of this study, and rucaparib and BMN-673 were selected because they are potent PARP trappers [22, 23]. We screened four EWSCs (ES7, ES8, MHH-ES-1, A673), the ES8-derived PARPi-resistant line OLAR5, one *BRCA1*-mutant (MDA-MB-436) and two non-Ewing’s control lines (DU-145 and U-2-OS). EWSCs were very sensitive to camptothecin alone, and a combination with PARPi failed to enhance sensitivity at the doses tested (Fig 5A and S5 Fig). Similarly, EWSCs were very sensitive to cisplatin alone but some further sensitization was observed in combination with PARPi in some EWSC cell lines. Importantly, temozolomide more substantially enhanced sensitivity to PARPi in all EWSCs tested, doing so to a degree comparable with MMS (Fig 5A and S5 Fig). For example, whereas treatment with 0.5μM niraparib had little effect on EWSCs, combination with 200μM temozolomide led to an almost complete loss of cell viability in all EWSCs tested (Fig 5B). The enhanced sensitivity with temozolomide was observed with multiple PARPi (niraparib, rucaparib, olaparib and BMN-673) and in all EWSC lines tested (Fig 5B and S5 and S6A Figs). The combination of olaparib with temozolomide induced apoptosis within 48 hours (Fig 5C).

The enhanced sensitivity to PARPi in combination with temozolomide was not specific to EWSCs. In DU-145 cells we observed sensitivity comparable with EWSCs, and PARP inhibition marginally potentiated the effects of temozolomide treatment in U-2-OS cells (S6B Fig). By contrast, when compared with the parental line ES8, neither MMS nor temozolomide enhanced sensitivity to PARPi in the PARPi-resistant EWSC, OLAR5, which had down-regulated PARP1 expression (S6C Fig).

To determine whether temozolomide and PARP1 inhibition enhanced the trapping of PARP1, we used a cellular sub-fractionation assay. We were unable to detect an increase in PARP1-DNA complexes with PARPi alone, or at concentrations at which enhanced sensitivity was detected in viability assays, likely due to lack of sensitivity of the trapping assay. However, 1mM temozolomide and 10μM olaparib drove PARP1 trapping on chromatin to detectable levels, confirming that temozolomide enhanced PARP1 trapping by PARPi (Fig 5D, PARP1 lanes 6 and 10). The lack of sensitivity of the trapping assay is demonstrated by the fact that MMS strongly potentiated the effects of olaparib on cell viability at concentrations of 0.0001% and 0.5μM respectively (S6D Fig), whereas PARP1-DNA complexes were only detected at 10–100 fold higher concentrations (0.01% MMS and 5μM olaparib; Fig 5D and S6E Fig). In aggregate, these data suggested that the toxicity of PARPi in EWSCs is due to cytotoxic PARP1 trapping, and that the combination with DNA alkylating agents, such as MMS or temozolomide, likely enhances toxicity through increased PARP1 trapping.

### Temozolomide enhances PARP inhibitor sensitivity in multiple tumour types

**Fig 6:**
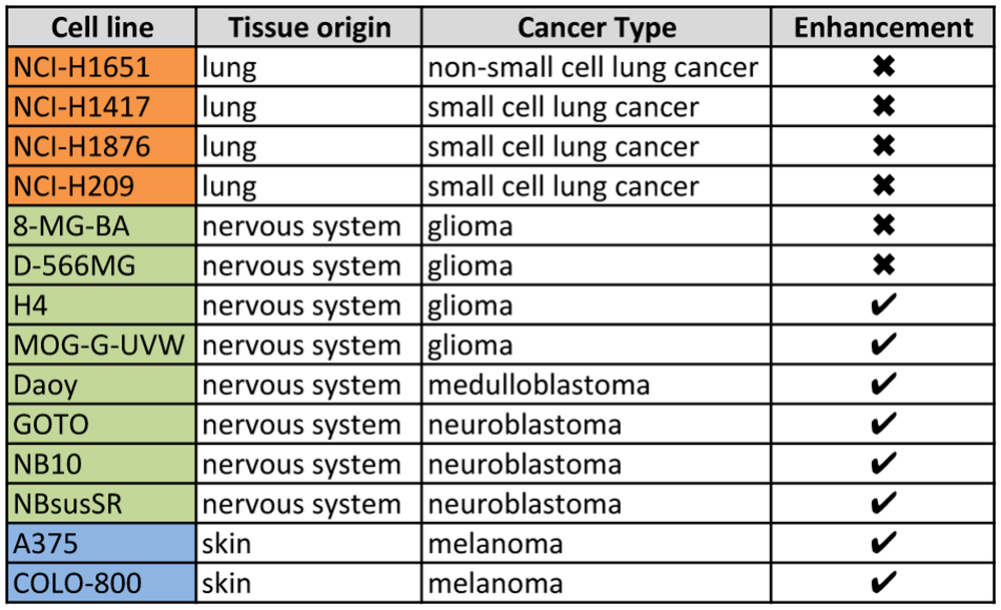
Temozolomide enhances PARP inhibitor sensitivity in multiple tumour types. List of cell lines screened against a combination of olaparib and temozolomide. Whether enhancement of PARP inhibitor sensitivity with temozolomide is observed (✔) or not (✖) is indicated.

Having observed that the enhanced effect of PARPi with temozolomide extended to non-EWSC cells, such as U-2-OS and DU-145, we re-analyzed our drug sensitivity data (S2 Data) to identify other cell lines that might be particularly sensitive to this combination. Thus, we identified cell lines with a similar drug sensitivity profile to EWSCs, in particular with IC_50_ values >1.5 standard deviations lower than the mean for olaparib, BMN-673 and camptothecin, and cross-sensitivity to at least two of these inhibitors. These criteria enriched for 42 non-EWSC cell lines (of 840 cell lines with a complete dataset; 6%) primarily from nervous system (glioma and neuroblastoma), lung, blood and ovary, and to a lesser extent cell lines from various other tissue types, such as melanoma (S2 Data). A subset of candidate cell lines (n = 14) was screened with a combination of olaparib with temozolomide, and enhanced sensitivity was observed in 6 of 8 nervous system cell lines and in both melanoma cell lines tested (Fig 6 and S7A Fig). By contrast, we observed at most additive effects in lung cell lines tested (4 of 4 lines). Thus, sensitivity to PARPi, enhanced by combination with temozolomide, may be prevalent in a subset of cells within multiple tumour types. Indeed, when we performed sub-fractionation assays in three nervous system and two melanoma cell lines, we detected PARP1-DNA complexes in all (S7B Fig, compare lanes 6 and 10). Interestingly, we detected trapped PARP1-DNA complexes in U251 glioma cells, which did not meet our drug sensitivity criteria and also did not have enhanced sensitivity to the combination of olaparib with temozolomide, indicating that PARP-DNA complexes are not toxic to all cells (S7C and S7D Fig, compare lanes 6 and 10).

## Discussion

PARP inhibition elicits anti-tumour activity in *BRCA*-mutant HR-deficient cancers [9–14], due to the dependency of these cancers on PARP1 activity in SSB repair to avoid replication-dependent accumulation of DSBs. Here, we confirm, using an expanded dataset, that EWSCs are hypersensitive to multiple PARPi chemotypes [31–35]. Olaparib treatment led to activation of DDR pathways and formation of RAD51 foci (a marker for functional HR), and depletion of HR proteins enhanced olaparib sensitivity. We did not identify aberrations by exome sequencing or western blotting in any of the established DDR proteins tested. Thus, although we are unable to exclude an underlying DNA repair defect, our results suggest that DSB repair by HR in EWSC lines is at least partially operative. This is consistent with an exceptionally low burden of mutations and structural variation in Ewing’s sarcoma patient tumours, which is in contrast to tumors deficient in key DNA repair processes [41, 43].

An alternative mechanism of toxicity for PARPi has been described, where inhibition of PARP blocks auto-PARylation and prevents PARP release from DNA [19–23]. We describe data supporting a model in which EWSCs are hypersensitive to PARP1 trapping. We demonstrated that an EWSC line with acquired resistance to olaparib had downregulated PARP1 protein, and siRNA-mediated depletion of PARP1 rescued EWSCs from PARPi hypersensitivity, indicating that PARP1 protein is required for drug toxicity. Recent reports have observed similar mechanisms of resistance to PARPi in other cell types [44, 45]. We hypothesized that combination with a chemotherapy agent would drive accumulation of DNA damage in EWSCs, heightening the recruitment of PARP1 to DNA for SSB repair, and thereby driving enhanced PARP1 trapping. Cisplatin did not increase sensitivity to PARPi in EWSC, whereas sensitivity was enhanced with DNA alkylating agents temozolomide and MMS. This is consistent with a very recent report by Murai *et al*, published while our studies were on-going [46]. Temozolomide with PARPi increased PARP1 trapping to levels detectable by biochemical assays and enhanced activation of apoptosis in EWSCs. Since OLAR5 cells, which had downregulated PARP1 were not hypersensitive to combination of MMS or temozolomide with PARPi, our data strongly suggest that sensitivity is the result of enhanced PARP1 trapping, likely as a result of recruitment of PARP1 to DNA in adduct-repair of lesions driven by MMS or temozolomide [42]. Nevertheless, we cannot rule out that enhanced sensitivity is a result of the combined toxicities of DNA lesions caused by MMS and temozolomide, and PARP1 trapping.

Our drug-combination screen and previous studies have demonstrated that DU-145 prostate cancer cells are very sensitive to the combination of temozolomide with PARP inhibition, and PARP trapping has been demonstrated in these cells [22, 23]. In this current study, we revealed exquisite sensitivity to the combination of temozolomide with olaparib across multiple cell lines from different tumour types. PARP trapping may thus be a more general mechanism of sensitivity to PARPi than so far recognised, potentially extending PARPi use to patients of multiple tumour types, provided that a biomarker for trapping sensitivity can be identified.

EWSCs highly express PARP1 both at the mRNA and protein level, with its expression suggested to be directly regulated by the EWS-FLI1 fusion protein [47, 48], and EWS-FLI1 expression induces DNA damage when overexpressed in PC-3 prostate cells [24, 48]. It has been proposed that olaparib hypersensitivity of EWSCs is due to a combined effect of potentiated levels of DNA damage and disruption of EWS-FLI1 transcriptional activity [24]. Our data support olaparib potentiating DNA damage through PARP1 trapping, and although we did not directly assess the effect of PARP inhibition on the EWS-FLI1 transcriptional programme, our results implicate PARP1 trapping and DNA damage in S-phase as the primary mechanism of toxicity. By inhibiting cell cycle progression or replication, olaparib-induced DSB breaks are prevented. Moreover, veliparib, which is a potent inhibitor of PARP enzymatic activity but a poor trapper, was less toxic in EWSC cells [22]. Thus, we propose that the EWS-FLI1 transcriptional program primes EWSCs for hypersensitivity to PARPi by inducing high PARP1 expression, increasing the availability of PARP1 for trapping, and elevating basal DNA damage. We hypothesise that elevated basal DNA damage is managed by a primed DDR but that disruption of the equilibrium between DNA damage and repair, either through PARP1 trapping or agents driving cytotoxic DNA lesions, triggers apoptosis. Moreover, it is possible that EWSCs in particular, are less able to process trapped PARP1 or toxic DSBs at replication forks, making them distinctively more sensitive to PARP trapping than other cell lines. The mechanism by which this may occur, however, remains to be elucidated.

When olaparib was tested as a single-agent in 12 adult Ewing’s sarcoma patients with recurrent disease as part of a phase II clinical trial, no partial or complete responses were observed [25, 27]. This is consistent with the minimal activity of single-agent PARPi in Ewing’s sarcoma xenografts, suggesting that PARPi may not trap PARP as efficiently *in vivo* [24, 28]. Our results provide mechanistic insights that support on-going trials combining temozolomide with olaparib in Ewing’s sarcoma patients, a combination already validated in xenograft and orthotopic models [27–29, 49, 50]. Combination of PARPi with temozolomide would thus be predicted to enhance the level of PARP1 trapping in Ewing’s sarcoma tumors to achieve greater clinical efficacy.

First-line therapy in Ewing’s sarcoma patients currently consists of a five-drug regimen of vincristine, doxorubicin, cyclophosphamide, ifosfamide and etoposide. Cyclophosphamide and ifosfamide are, like temozolomide, alkylating agents driving lesions likely to be repaired by PARP1. Thus combination of these agents with PARPi could result in more effective therapy provided that this combination is tolerated in patients. If so, combining etoposide with alkylating agents and PARP1 inhibition would potentially suppress resistance mediated through down-regulation of PARP1, which we have shown can provide a possible route of resistance to this type of combination therapy.

Our study suggests that PARP1 inhibition should be evaluated in combination with the standard-of-care multi-chemotherapy regimen to assess its ability to improve treatment outcomes in Ewing’s sarcoma, with a view to at least delay the onset of recurrent disease. Thus, our results provide a mechanistic framework to understand the activity of PARPi in EWSCs, which should help promote the successful development of a targeted therapy for the treatment of Ewing’s sarcoma.

## Supporting Information

S1 Data Table of cell line drug sensitivity data. (XLSX)

S2 Data Table of cell lines with cross-sensitivity to BMN-673, olaparib and camptothecin. (XLSX)

S1 Fig Sensitivity of EWSCs to DNA-damaging agents. **(A)** List of Ewing’s sarcoma cell lines in which disruption of the EWS gene was confirmed (✔), undetected (✖) or not determined (ND) by either FISH, PCR or RNA-sequencing. **(B)** Scatter plots of IC50 (μM) values on a log scale comparing drug sensitivity of EWS-FLI1-translocation-positive and wild-type (WT) cell lines to various S-phase damaging agents. Each circle represents the IC50 of one cell line and the red bar is the geometric mean. The sample size (n) is indicated below each plot and the drug name above along with the significance of the association as determined by an unpaired two-sample t-test.
(TIF)

S2 Fig DDR proteins are expressed in EWSCs. **(A)** Expression levels of DDR proteins in parental ES8 and PARP inhibitor-resistant OLAR5 cells. Tubulin served as a loading control. **(B)** Expression of BRCA1 and BRCA2 in BRCA1, BRCA2 and negative control IgG immunoprecipitates (IP) from Ewing’s (ES7, ES8, MHH-ES-1) and control cell lines. 5% of whole cell lysates were western blotted (WB) for tubulin to control for variations in IP volume (input).
(TIF)

S3 Fig The DDR and HR are functional in EWSCs. **(A)** MHH-ES-1 cells were treated with olaparib for 16 hours following a 6-hour pre-treatment with palbociclib (CDK4/6i) or vehicle and percentage of γH2AX responders determined. **(B)** ES8 cells were treated with vehicle or olaparib and stained with Hoechst (nucleus; blue) and for 53BP1 (green). Images on the left are of the 8-hour time point. The graph measures fold increase in 53BP1 responders at the time points indicated. **(C)** MHH-ES-1 and ES7 cells were treated with vehicle, 5μM aphidicolin (AphD), 5μM olaparib (Ola) or a 30-minute pre-treatment with aphidicolin followed by olaparib for 8 hours and percentage of γH2AX responders determined. Asterisks indicate *student’s paired t-test P* value ** (P<0.01), ***(P<0.001), ****(P<0.0001), ns = not significant, relative to control. **(D)** ES8 cells were labeled with EdU and treated with vehicle or olaparib before fixing and staining for DAPI (grey, nucleus), RAD51 (green), γH2AX (red) and EdU incorporation (blue, S-phase cells) as indicated. Scale bars are 500μm in size in rows 1–2 and 100μm in row 3. Error bars represent the standard deviation of the mean of technical triplicates and results are representative of 3 independent experiments.
(TIF)

S4 Fig EWSCs are sensitive to PARP1 trapping. **(A)** Western blot of ES7 and MHH-ES-1 cells treated with olaparib for the times indicated. Markers are grouped as part of ATM or ATR signaling. Tubulin served as a loading control. **(B)** Expression levels of PARP1 in cells transfected with two distinct PARP1 siRNAs (1 and 2) or a scrambled control. **(C)** Relative viability of mock-transfected and PARP1_1 siRNA-transfected ES8 cells treated with vehicle or rucaparib. Asterisks indicate *student’s paired t-test P* value, ***P<0.001, ns = not significant. **(D)** Relative viability of mock-transfected and PARP1_1 siRNA-transfected ES7 and MHH-ES-1 cells treated with vehicle, olaparib or rucaparib. Asterisks indicate *student’s paired t-test P* value **P<0.01, ns = not significant. **(E)** Relative viability of mock-transfected and PARP1 siRNA(1 and 2)-transfected cells treated with vehicle or olaparib. Viability values are the mean of technical duplicates.
(TIF)

S5 Fig Combination drug screening results. Ewing’s cells (ES7, A673, MHH-ES-1 and ES8), the ES8-derived PARP inhibitor-resistant OLAR5 cells, non-Ewing’s control lines (U-2-OS, DU-145) and a BRCA1-mutant breast cancer cell line (MDA-MB-436) were screened against titrated concentrations of three different PARP inhibitors (niraparib, rucaparib, BMN-673) in combination with titrated concentrations of three chemotherapies (camptothecin, cisplatin and temozolomide) or methyl methanesulfonate (MMS). Heatmaps show relative viability values of each combination. The PARP inhibitor and relevant concentrations are on the vertical axis and the chemotherapy or MMS and relevant concentrations are on the horizontal axes. Drug concentrations are in micromolar (μM) with high viability values in red and low viability values in green. Viability values are the mean of technical duplicates.
(PDF)

S6 Fig Temozolomide enhances PARP inhibitor sensitivity in cancer cells. **(A)** Relative viability of ES8, ES7 and MHH-ES-1 cells treated with temozolomide (log scale) in the presence or absence of olaparib. Relative viability is normalized to 0.5μM olaparib and dotted lines indicate the viability of olaparib-only controls. Error bars represent the standard deviation of the mean of technical triplicates. **(B)** Relative viability of U-2-OS and DU-145 cells treated with vehicle, niraparib or temozolomide (TMZ) alone, or in combination. The combination effect is highlighted in green. **(C)** Heatmap of relative viability values of OLAR5 cells against a combination of niraparib and temozolomide (TMZ). High viability values are in red and low viability values in green. Graph shows the corresponding dose response curves measuring relative viability with a separate line plotted for each concentration of the combined drug. The dose response for niraparib alone is highlighted in red. Viability values are the mean of technical duplicates. **(D)** Relative viability of ES8, ES7 and MHH-ES-1 cells treated with MMS (log scale) in the presence or absence of olaparib. Relative viability is normalized to 0.5μM olaparib and dotted lines indicate the viability of olaparib-only controls. Error bars represent the standard deviation of the mean of technical triplicates. **(E)** MHH-ES-1 cells were treated with vehicle (-) or a titration of MMS in combination with olaparib for 2 hours. A cellular sub-fractionation assay was performed and soluble nuclear and chromatin fractions western blotted for PARP1. Histone-3 (H3) served as a fractionation control.
(TIF)

S7 Fig PARPi and temozolomide synergise in glioma, neuroblastoma and melanoma cells. **(A)** Relative viability of cells treated with temozolomide (log scale) in the presence or absence of olaparib for 72 hours, or 6 days where indicated. Data represent technical duplicates. **(B)** Cellular sub-fractionation assay following treatment of cells with vehicle (-), MMS in combination with olaparib (ola), or olaparib and temozolomide (TMZ) alone or in combination for 4 hours. **(C)** Relative viability of U251 cells treated with temozolomide (log scale) in the presence or absence of olaparib for 6 days. **(D)** A cellular sub-fractionation assay in U251 cells.
(TIF)

S1 Text Supplementary methods. (DOCX)
