# Supplementary figures and images for: Combinations of PARP Inhibitors with Temozolomide Drive PARP1 Trapping and Apoptosis in Ewing’s Sarcoma
Source: PLoS One. 2015 Oct 27;10(10):e0140988. doi: 10.1371/journal.pone.0140988 (PMC4624427; doi:10.1371/journal.pone.0140988)

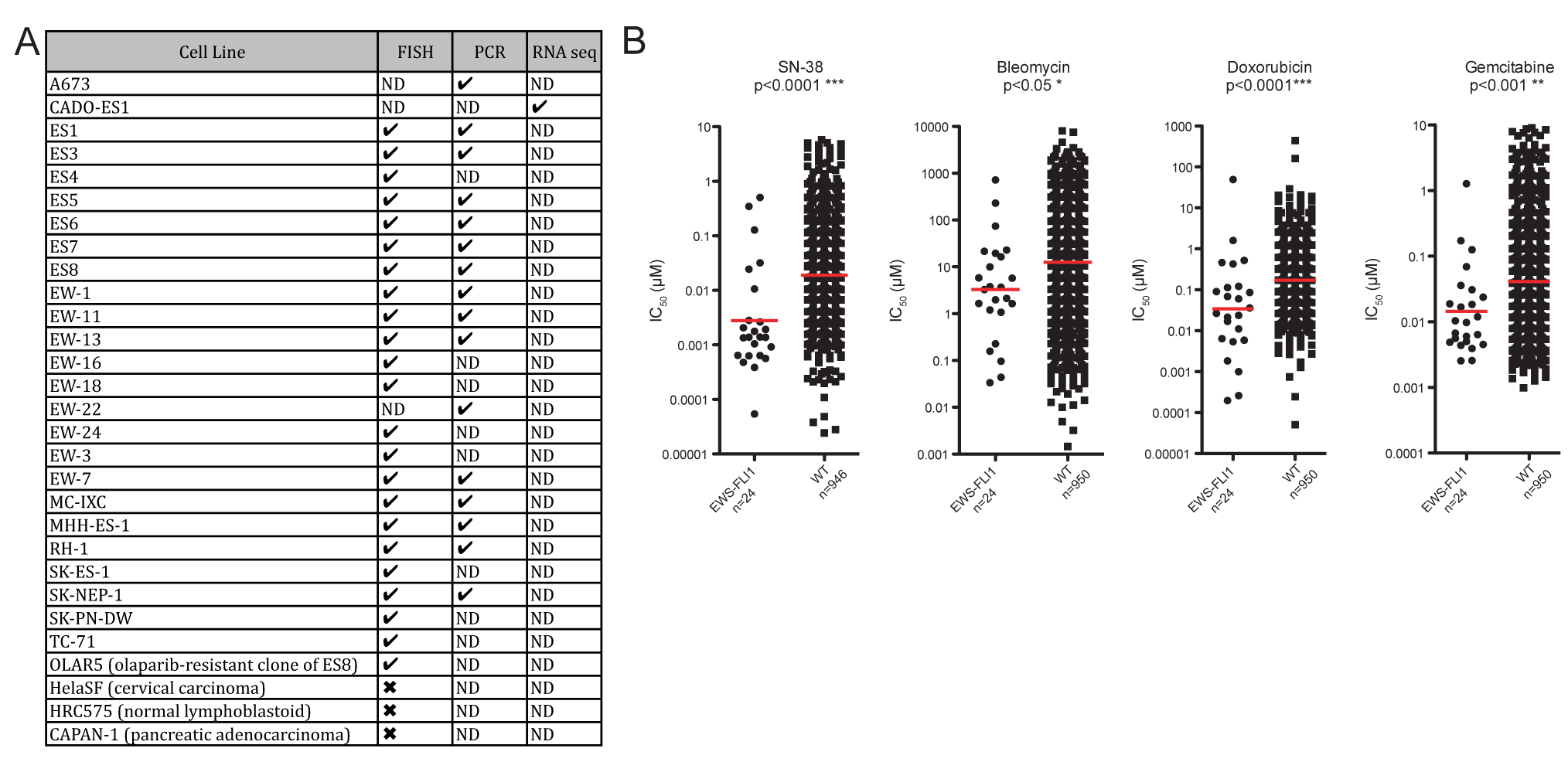

Supplement: S1 Fig — (A) List of Ewing’s sarcoma cell lines in which disruption of the EWS gene was confirmed (✔), undetected (✖) or not determined (ND) by either FISH, PCR or RNA-sequencing. (B) Scatter plots of IC50 (μM) values on a log scale comparing drug sensitivity of EWS-FLI1-translocation-positive and wild-type (WT) cell lines to various S-phase damaging agents. Each circle represents the IC50 of one cell line and the red bar is the geometric mean. The sample size (n) is indicated below each plot and the drug name above along with the significance of the association as determined by an unpaired two-sample t-test. (TIF) [file pone.0140988.s003.tif]

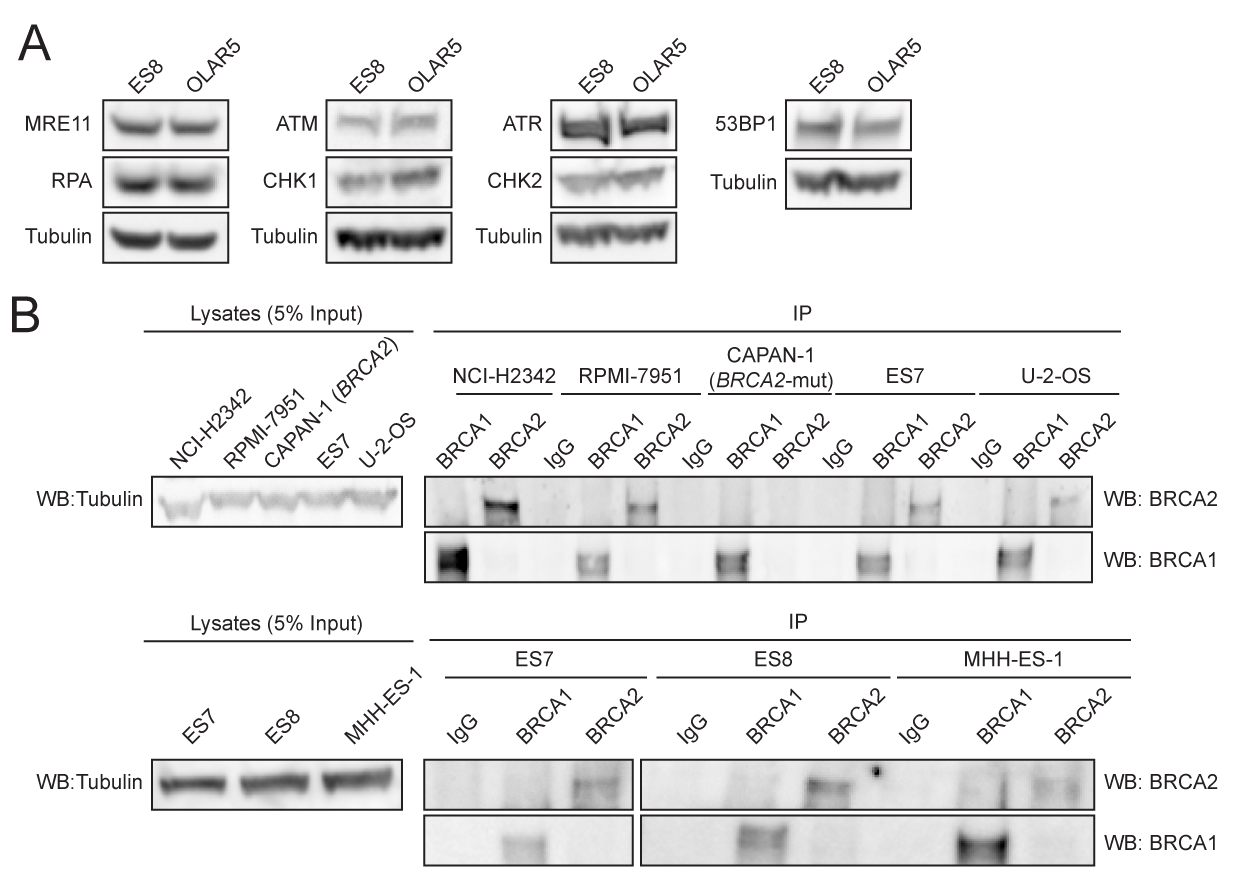

Supplement: S2 Fig — (A) Expression levels of DDR proteins in parental ES8 and PARP inhibitor-resistant OLAR5 cells. Tubulin served as a loading control. (B) Expression of BRCA1 and BRCA2 in BRCA1, BRCA2 and negative control IgG immunoprecipitates (IP) from Ewing’s (ES7, ES8, MHH-ES-1) and control cell lines. 5% of whole cell lysates were western blotted (WB) for tubulin to control for variations in IP volume (input). (TIF) [file pone.0140988.s004.tif]

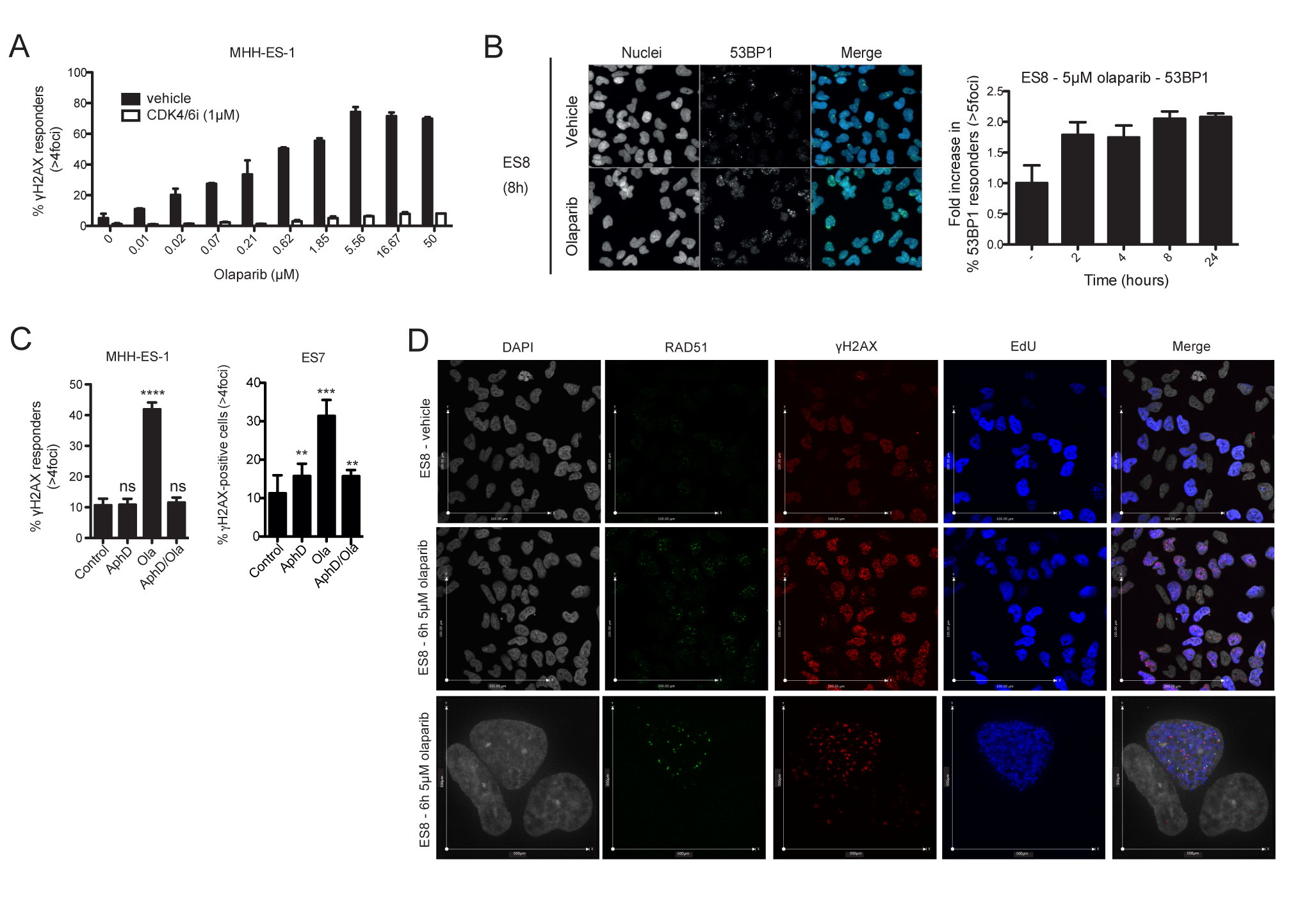

Supplement: S3 Fig — (A) MHH-ES-1 cells were treated with olaparib for 16 hours following a 6-hour pre-treatment with palbociclib (CDK4/6i) or vehicle and percentage of γH2AX responders determined. (B) ES8 cells were treated with vehicle or olaparib and stained with Hoechst (nucleus; blue) and for 53BP1 (green). Images on the left are of the 8-hour time point. The graph measures fold increase in 53BP1 responders at the time points indicated. (C) MHH-ES-1 and ES7 cells were treated with vehicle, 5μM aphidicolin (AphD), 5μM olaparib (Ola) or a 30-minute pre-treatment with aphidicolin followed by olaparib for 8 hours and percentage of γH2AX responders determined. Asterisks indicate student’s paired t-test P value ** (P<0.01), ***(P<0.001), ****(P<0.0001), ns = not significant, relative to control. (D) ES8 cells were labeled with EdU and treated with vehicle or olaparib before fixing and staining for DAPI (grey, nucleus), RAD51 (green), γH2AX (red) and EdU incorporation (blue, S-phase cells) as indicated. Scale bars are 500μm in size in rows 1–2 and 100μm in row 3. Error bars represent the standard deviation of the mean of technical triplicates and results are representative of 3 independent experiments. (TIF) [file pone.0140988.s005.tif]

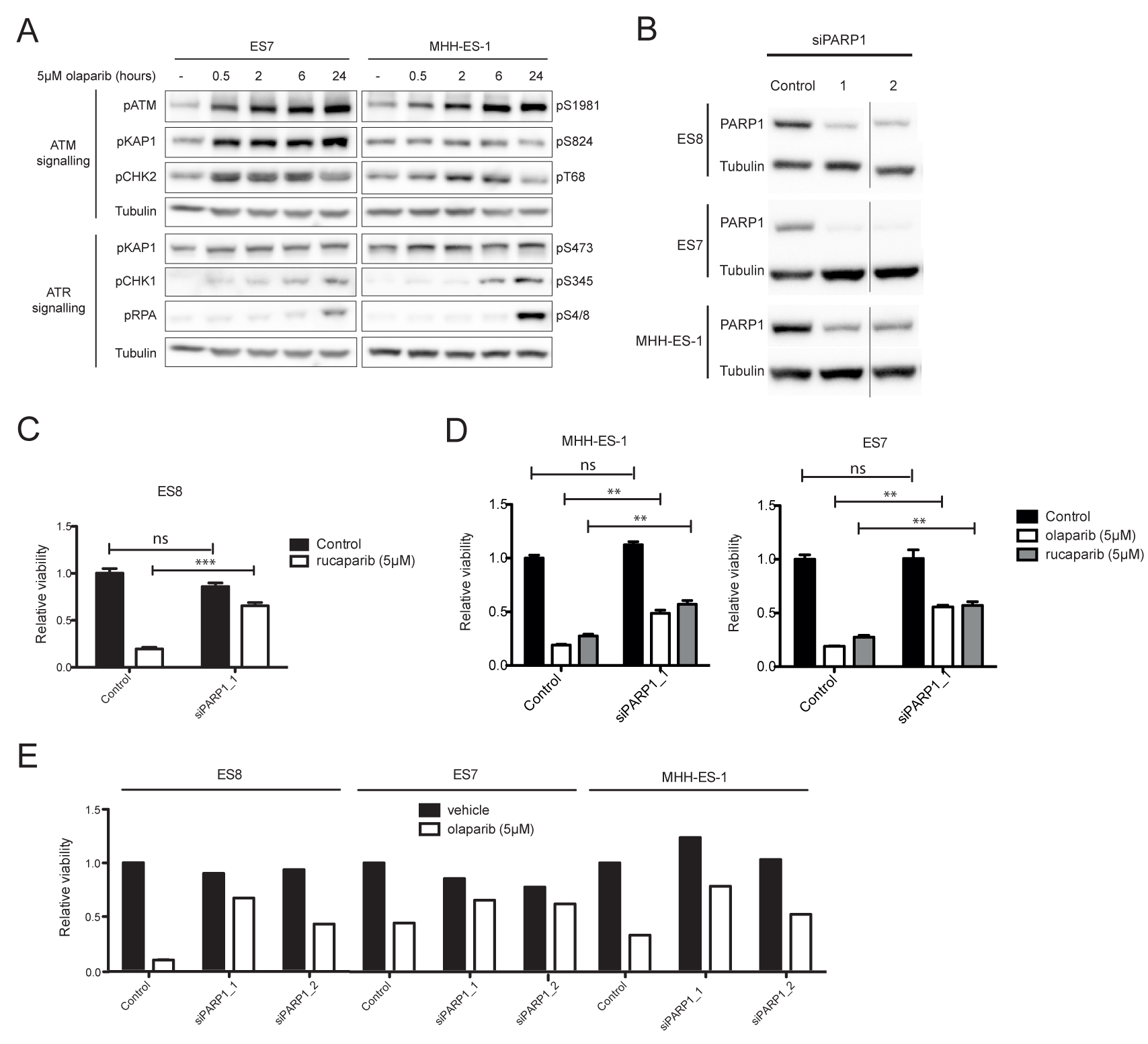

Supplement: S4 Fig — (A) Western blot of ES7 and MHH-ES-1 cells treated with olaparib for the times indicated. Markers are grouped as part of ATM or ATR signaling. Tubulin served as a loading control. (B) Expression levels of PARP1 in cells transfected with two distinct PARP1 siRNAs (1 and 2) or a scrambled control. (C) Relative viability of mock-transfected and PARP1_1 siRNA-transfected ES8 cells treated with vehicle or rucaparib. Asterisks indicate student’s paired t-test P value, ***P<0.001, ns = not significant. (D) Relative viability of mock-transfected and PARP1_1 siRNA-transfected ES7 and MHH-ES-1 cells treated with vehicle, olaparib or rucaparib. Asterisks indicate student’s paired t-test P value **P<0.01, ns = not significant. (E) Relative viability of mock-transfected and PARP1 siRNA(1 and 2)-transfected cells treated with vehicle or olaparib. Viability values are the mean of technical duplicates. (TIF) [file pone.0140988.s006.tif]

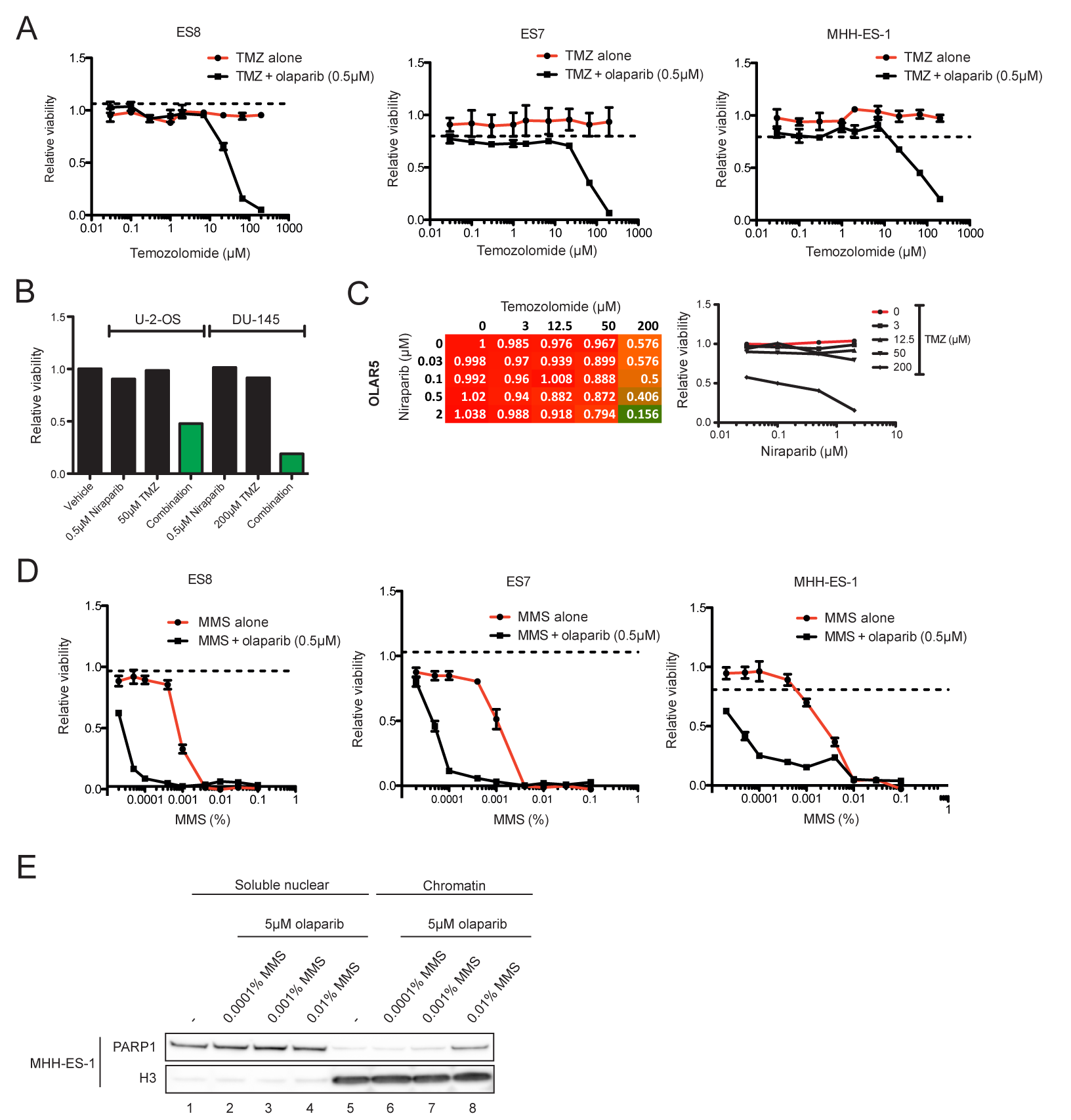

Supplement: S6 Fig — (A) Relative viability of ES8, ES7 and MHH-ES-1 cells treated with temozolomide (log scale) in the presence or absence of olaparib. Relative viability is normalized to 0.5μM olaparib and dotted lines indicate the viability of olaparib-only controls. Error bars represent the standard deviation of the mean of technical triplicates. (B) Relative viability of U-2-OS and DU-145 cells treated with vehicle, niraparib or temozolomide (TMZ) alone, or in combination. The combination effect is highlighted in green. (C) Heatmap of relative viability values of OLAR5 cells against a combination of niraparib and temozolomide (TMZ). High viability values are in red and low viability values in green. Graph shows the corresponding dose response curves measuring relative viability with a separate line plotted for each concentration of the combined drug. The dose response for niraparib alone is highlighted in red. Viability values are the mean of technical duplicates. (D) Relative viability of ES8, ES7 and MHH-ES-1 cells treated with MMS (log scale) in the presence or absence of olaparib. Relative viability is normalized to 0.5μM olaparib and dotted lines indicate the viability of olaparib-only controls. Error bars represent the standard deviation of the mean of technical triplicates. (E) MHH-ES-1 cells were treated with vehicle (-) or a titration of MMS in combination with olaparib for 2 hours. A cellular sub-fractionation assay was performed and soluble nuclear and chromatin fractions western blotted for PARP1. Histone-3 (H3) served as a fractionation control. (TIF) [file pone.0140988.s008.tif]

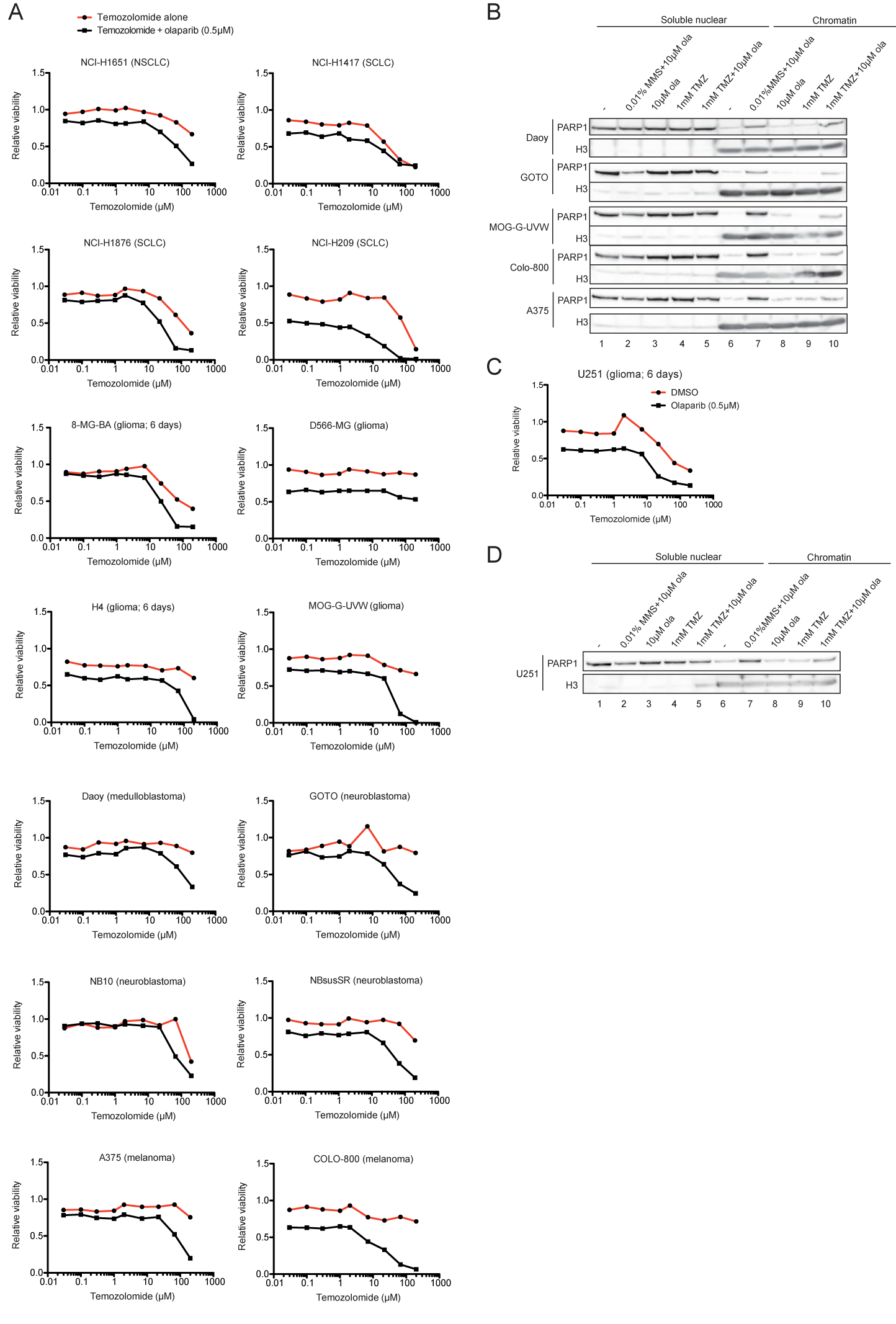

Supplement: S7 Fig — (A) Relative viability of cells treated with temozolomide (log scale) in the presence or absence of olaparib for 72 hours, or 6 days where indicated. Data represent technical duplicates. (B) Cellular sub-fractionation assay following treatment of cells with vehicle (-), MMS in combination with olaparib (ola), or olaparib and temozolomide (TMZ) alone or in combination for 4 hours. (C) Relative viability of U251 cells treated with temozolomide (log scale) in the presence or absence of olaparib for 6 days. (D) A cellular sub-fractionation assay in U251 cells. (TIF) [file pone.0140988.s009.tif]
