# Supplementary material for: Combinations of PARP Inhibitors with Temozolomide Drive PARP1 Trapping and Apoptosis in Ewing’s Sarcoma
Source: PLoS One. 2015 Oct 27;10(10):e0140988. doi: 10.1371/journal.pone.0140988 (PMC4624427; doi:10.1371/journal.pone.0140988)
Supplement: S5 Fig — Ewing’s cells (ES7, A673, MHH-ES-1 and ES8), the ES8-derived PARP inhibitor-resistant OLAR5 cells, non-Ewing’s control lines (U-2-OS, DU-145) and a BRCA1-mutant breast cancer cell line (MDA-MB-436) were screened against titrated concentrations of three different PARP inhibitors (niraparib, rucaparib, BMN-673) in combination with titrated concentrations of three chemotherapies (camptothecin, cisplatin and temozolomide) or methyl methanesulfonate (MMS). Heatmaps show relative viability values of each combination. The PARP inhibitor and relevant concentrations are on the vertical axis and the chemotherapy or MMS and relevant concentrations are on the horizontal axes. Drug concentrations are in micromolar (μM) with high viability values in red and low viability values in green. Viability values are the mean of technical duplicates. (PDF) [file pone.0140988.s007.pdf]

Niraparib/Camptothecin

ES7

Niraparib (μM) \ Camptothecin (μM)	0	0.002	0.006	0.03	0.1
0	1	0.066	0.023	0.020	0.015
0.03	0.999	0.041	0.023	0.018	0.018
0.1	0.925	0.032	0.021	0.020	0.016
0.5	0.710	0.024	0.018	0.018	0.015
2	0.072	0.025	0.020	0.021	0.021

U-2-OS

Niraparib (μM) \ Camptothecin (μM)	0	0.002	0.006	0.03	0.1
0	1	0.828	0.502	0.442	0.410
0.03	1.046	0.841	0.497	0.450	0.386
0.1	1.038	0.749	0.459	0.433	0.328
0.5	1.036	0.703	0.470	0.398	0.354
2	0.905	0.592	0.442	0.423	0.341

A673

Niraparib (μM) \ Camptothecin (μM)	0	0.002	0.006	0.03	0.1
0	1	0.279	0.118	0.064	0.046
0.03	1.001	0.206	0.099	0.055	0.040
0.1	0.938	0.170	0.085	0.056	0.041
0.5	0.786	0.170	0.084	0.048	0.038
2	0.363	0.123	0.067	0.053	0.044

DU-145

Niraparib (μM) \ Camptothecin (μM)	0	0.002	0.006	0.03	0.1
0	1	0.865	0.242	0.141	0.104
0.03	1.014	0.852	0.233	0.132	0.106
0.1	0.976	0.824	0.206	0.125	0.090
0.5	1.015	0.639	0.180	0.123	0.097
2	0.893	0.414	0.181	0.121	0.093

MHH-ES-1

Niraparib (μM) \ Camptothecin (μM)	0	0.002	0.006	0.03	0.1
0	1	0.186	0.052	0.021	0.015
0.03	0.985	0.138	0.037	0.022	0.020
0.1	0.919	0.120	0.038	0.025	0.015
0.5	0.728	0.096	0.034	0.025	0.022
2	0.201	0.067	0.036	0.024	0.021

MDA-MB-436

Niraparib (μM) \ Camptothecin (μM)	0	0.002	0.006	0.03	0.1
0	1	0.714	0.449	0.351	0.305
0.03	1.010	0.659	0.403	0.400	0.280
0.1	0.938	0.586	0.426	0.327	0.288
0.5	0.894	0.584	0.421	0.317	0.267
2	0.677	0.523	0.388	0.347	0.295

ES8

Niraparib (μM) \ Camptothecin (μM)	0	0.002	0.006	0.03	0.1
0	1	0.064	0.015	0.012	0.011
0.03	1.000	0.022	0.014	0.013	0.013
0.1	0.973	0.019	0.012	0.010	0.012
0.5	0.816	0.018	0.013	0.012	0.012
2	0.031	0.011	0.014	0.011	0.015

OLAR5

Niraparib (μM) \ Camptothecin (μM)	0	0.002	0.006	0.03	0.1
0	1	0.097	0.044	0.035	0.032
0.03	0.998	0.107	0.045	0.034	0.027
0.1	0.992	0.098	0.050	0.033	0.029
0.5	1.020	0.095	0.046	0.039	0.029
2	1.038	0.089	0.047	0.035	0.029

Niraparib/Cisplatin

ES7

Niraparib (μM) \ Cisplatin (μM)	0	0.2	0.6	2.5	10
0	1	0.888	0.493	0.087	0.034
0.03	0.999	0.846	0.469	0.092	0.033
0.1	0.925	0.693	0.382	0.091	0.032
0.5	0.710	0.464	0.238	0.073	0.029
2	0.072	0.055	0.056	0.043	0.027

U-2-OS

Niraparib (μM) \ Cisplatin (μM)	0	0.2	0.6	2.5	10
0	1	1.100	0.900	0.600	0.300
0.03	1.000	1.000	0.900	0.600	0.300
0.1	1.000	1.000	0.900	0.500	0.300
0.5	1.000	1.000	0.800	0.500	0.300
2	0.900	0.800	0.700	0.500	0.200

A673

Niraparib (μM) \ Cisplatin (μM)	0	0.2	0.6	2.5	10
0	1	0.843	0.416	0.147	0.027
0.03	1.001	0.793	0.390	0.156	0.028
0.1	0.938	0.696	0.402	0.138	0.028
0.5	0.786	0.613	0.333	0.144	0.027
2	0.363	0.296	0.238	0.138	0.029

DU-145

Niraparib (μM) \ Cisplatin (μM)	0	0.2	0.6	2.5	10
0	1	0.889	0.831	0.440	0.105
0.03	1.014	0.994	0.842	0.446	0.101
0.1	0.976	1.019	0.793	0.414	0.103
0.5	1.015	0.989	0.836	0.358	0.109
2	0.893	0.885	0.696	0.295	0.106

MHH-ES-1

Niraparib (μM) \ Cisplatin (μM)	0	0.2	0.6	2.5	10
0	1	0.968	0.870	0.493	0.160
0.03	0.985	0.966	0.835	0.461	0.156
0.1	0.919	0.909	0.774	0.429	0.168
0.5	0.728	0.649	0.531	0.304	0.137
2	0.201	0.183	0.181	0.156	0.100

MDA-MB-436

Niraparib (μM) \ Cisplatin (μM)	0	0.2	0.6	2.5	10
0	1	0.798	0.529	0.461	0.190
0.03	1.010	0.795	0.547	0.475	0.184
0.1	0.938	0.805	0.582	0.420	0.166
0.5	0.894	0.720	0.549	0.418	0.189
2	0.677	0.649	0.518	0.418	0.220

ES8

Niraparib (μM) \ Cisplatin (μM)	0	0.2	0.6	2.5	10
0	1	0.980	0.736	0.057	0.013
0.03	1.000	0.933	0.637	0.056	0.014
0.1	0.973	0.877	0.504	0.050	0.020
0.5	0.816	0.575	0.172	0.031	0.014
2	0.031	0.028	0.023	0.023	0.015

OLAR5

Niraparib (μM) \ Cisplatin (μM)	0	0.2	0.6	2.5	10
0	1	0.960	0.949	0.334	0.079
0.03	0.998	0.926	0.929	0.344	0.084
0.1	0.992	0.994	0.891	0.337	0.090
0.5	1.020	0.944	0.971	0.331	0.092
2	1.038	0.951	0.894	0.364	0.095

Niraparib/Temozolomide

ES7

Niraparib (μM) \ Temozolomide (μM)	0	3	12.5	50	200
0	1	0.910	0.941	0.912	0.574
0.03	0.999	0.902	0.871	0.682	0.122
0.1	0.925	0.785	0.706	0.294	0.038
0.5	0.710	0.643	0.360	0.058	0.022
2	0.072	0.066	0.030	0.020	0.017

U-2-OS

Niraparib (μM) \ Temozolomide (μM)	0	3	12.5	50	200
0	1	1.015	0.970	0.986	0.664
0.03	1.046	1.064	0.988	0.944	0.554
0.1	1.038	1.074	0.979	0.865	0.489
0.5	1.036	1.083	1.007	0.715	0.395
2	0.905	0.878	0.610	0.478	0.401

A673

Niraparib (μM) \ Temozolomide (μM)	0	3	12.5	50	200
0	1	1.037	1.028	0.986	0.814
0.03	1.001	1.005	0.963	0.771	0.350
0.1	0.938	0.963	0.856	0.584	0.190
0.5	0.786	0.798	0.607	0.285	0.105
2	0.363	0.328	0.229	0.137	0.073

DU-145

Niraparib (μM) \ Temozolomide (μM)	0	3	12.5	50	200
0	1	0.874	0.995	0.956	0.916
0.03	1.014	1.053	1.060	0.922	0.840
0.1	0.976	0.968	0.937	0.977	0.510
0.5	1.015	0.883	0.919	0.709	0.189
2	0.893	0.888	0.784	0.329	0.138

MHH-ES-1

Niraparib (μM) \ Temozolomide (μM)	0	3	12.5	50	200
0	1	0.906	0.865	0.934	0.822
0.03	0.985	0.884	0.894	0.843	0.373
0.1	0.919	0.914	0.886	0.613	0.143
0.5	0.728	0.691	0.558	0.212	0.052
2	0.201	0.196	0.123	0.059	0.028

MDA-MB-436

Niraparib (μM) \ Temozolomide (μM)	0	3	12.5	50	200
0	1	1.048	0.946	0.979	0.767
0.03	1.010	1.064	0.882	0.854	0.617
0.1	0.938	0.886	0.894	0.755	0.475
0.5	0.894	0.817	0.765	0.520	0.385
2	0.677	0.703	0.604	0.437	0.338

ES8

Niraparib (μM) \ Temozolomide (μM)	0	3	12.5	50	200
0	1	1.002	0.962	0.914	0.781
0.03	1.000	1.073	0.950	0.726	0.071
0.1	0.973	1.003	0.854	0.281	0.014
0.5	0.816	0.691	0.345	0.029	0.015
2	0.031	0.026	0.018	0.015	0.014

OLAR5

Niraparib (μM) \ Temozolomide (μM)	0	3	12.5	50	200
0	1	0.985	0.976	0.967	0.576
0.03	0.998	0.970	0.939	0.899	0.576
0.1	0.992	0.960	1.008	0.888	0.500
0.5	1.020	0.940	0.882	0.872	0.406
2	1.038	0.988	0.918	0.794	0.156

Niraparib/MMS

ES7

Niraparib (μM) \ MMS (μM)	0	8	31	125	500
0	1	0.957	0.956	0.521	0.036
0.03	0.937	0.687	0.287	0.047	0.018
0.1	0.948	0.248	0.056	0.026	0.022
0.5	0.595	0.091	0.037	0.017	0.024
2	0.089	0.030	0.029	0.024	0.022

U-2-OS

Niraparib (μM) \ MMS (μM)	0	8	31	125	500
0	1	1.053	1.021	0.851	0.426
0.03	1.013	0.983	0.894	0.599	0.362
0.1	1.046	0.959	0.791	0.514	0.370
0.5	0.941	0.851	0.598	0.462	0.323
2	0.849	0.586	0.490	0.487	0.288

A673

Niraparib (μM) \ MMS (μM)	0	8	31	125	500
0	1	1.018	0.905	0.685	0.149
0.03	1.002	0.756	0.532	0.199	0.110
0.1	0.893	0.556	0.305	0.162	0.095
0.5	0.724	0.324	0.185	0.107	0.082
2	0.399	0.164	0.115	0.092	0.080

DU-145

Niraparib (μM) \ MMS (μM)	0	8	31	125	500
0	1	1.004	0.978	0.976	0.444
0.03	1.046	0.949	0.948	0.459	0.126
0.1	1.015	0.984	0.712	0.218	0.105
0.5	0.990	0.752	0.285	0.184	0.098
2	0.809	0.259	0.194	0.151	0.109

MHH-ES-1

Niraparib (μM) \ MMS (μM)	0	8	31	125	500
0	1	1.025	0.993	0.738	0.179
0.03	1.057	0.864	0.519	0.172	0.093
0.1	0.899	0.443	0.226	0.090	0.075
0.5	0.648	0.228	0.107	0.059	0.065
2	0.217	0.063	0.049	0.042	0.061

MDA-MB-436

Niraparib (μM) \ MMS (μM)	0	8	31	125	500
0	1	0.931	0.953	0.810	0.289
0.03	0.994	0.894	0.873	0.495	0.261
0.1	0.901	0.748	0.594	0.412	0.222
0.5	0.873	0.689	0.499	0.379	0.236
2	0.715	0.490	0.474	0.358	0.209

ES8

Niraparib (μM) \ MMS (μM)	0	8	31	125	500
0	1	1.010	1.015	0.721	0.025
0.03	0.964	0.762	0.212	0.026	0.017
0.1	0.913	0.223	0.033	0.016	0.020
0.5	0.648	0.026	0.018	0.015	0.014
2	0.045	0.020	0.020	0.022	0.016

OLAR5

Niraparib (μM) \ MMS (μM)	0	8	31	125	500
0	1	0.982	0.802	0.140	0.035
0.03	1.079	0.958	0.901	0.121	0.029
0.1	1.037	1.045	0.808	0.133	0.037
0.5	1.057	1.038	0.693	0.081	0.032
2	1.021	0.840	0.236	0.048	0.033

Rucaparib/Camptothecin

ES7

Rucaparib (μM) \ Camptothecin (μM)	0	0.002	0.006	0.03	0.1
0	1	0.061	0.023	0.019	0.017
0.08	0.942	0.030	0.021	0.019	0.019
0.3	0.841	0.028	0.024	0.020	0.017
1.2	0.688	0.026	0.019	0.016	0.016
5	0.163	0.027	0.023	0.017	0.017

U-2-OS

Rucaparib (μM) \ Camptothecin (μM)	0	0.002	0.006	0.03	0.1
0	1	0.770	0.451	0.406	0.370
0.08	0.987	0.580	0.446	0.385	0.305
0.3	1.022	0.560	0.405	0.352	0.298
1.2	0.979	0.508	0.412	0.379	0.329
5	0.837	0.445	0.427	0.388	0.373

A673

Rucaparib (μM) \ Camptothecin (μM)	0	0.002	0.006	0.03	0.1
0	1	0.243	0.094	0.050	0.033
0.08	0.988	0.164	0.070	0.037	0.033
0.3	0.958	0.144	0.070	0.043	0.033
1.2	0.856	0.145	0.073	0.046	0.031
5	0.526	0.139	0.074	0.051	0.043

DU-145

Rucaparib (μM) \ Camptothecin (μM)	0	0.002	0.006	0.03	0.1
0	1	0.915	0.201	0.103	0.096
0.08	1.036	0.629	0.149	0.108	0.091
0.3	1.077	0.612	0.153	0.104	0.088
1.2	0.974	0.485	0.149	0.114	0.535
5	0.895	0.300	0.132	0.104	0.084

MHH-ES-1

Rucaparib (μM) \ Camptothecin (μM)	0	0.002	0.006	0.03	0.1
0	1	0.178	0.049	0.027	0.020
0.08	0.936	0.124	0.041	0.021	0.019
0.3	0.922	0.112	0.045	0.023	0.017
1.2	0.709	0.094	0.038	0.024	0.016
5	0.312	0.084	0.043	0.026	0.021

MDA-MB-436

Rucaparib (μM) \ Camptothecin (μM)	0	0.002	0.006	0.03	0.1
0	1	0.650	0.424	0.323	0.267
0.08	0.886	0.558	0.403	0.305	0.259
0.3	0.883	0.521	0.363	0.285	0.251
1.2	0.869	0.522	0.376	0.314	0.260
5	0.727	0.514	0.397	0.362	0.295

ES8

Rucaparib (μM) \ Camptothecin (μM)	0	0.002	0.006	0.03	0.1
0	1	0.099	0.017	0.014	0.014
0.08	0.979	0.022	0.014	0.012	0.015
0.3	0.973	0.023	0.014	0.010	0.013
1.2	0.790	0.018	0.014	0.014	0.013
5	0.129	0.018	0.017	0.012	0.013

OLAR5

Rucaparib (μM) \ Camptothecin (μM)	0	0.002	0.006	0.03	0.1
0	1	0.117	0.059	0.043	0.034
0.08	1.044	0.107	0.052	0.042	0.033
0.3	1.032	0.108	0.063	0.041	0.034
1.2	1.046	0.119	0.056	0.048	0.044
5	1.040	0.127	0.077	0.062	0.046

Rucaparib/Cisplatin

ES7

Rucaparib (μM) \ Cisplatin (μM)	0	0.2	0.6	2.5	10
0	1	0.817	0.456	0.085	0.035
0.08	0.942	0.706	0.332	0.076	0.034
0.3	0.841	0.646	0.298	0.073	0.033
1.2	0.688	0.450	0.201	0.065	0.036
5	0.163	0.112	0.092	0.050	0.036

U-2-OS

Rucaparib (μM) \ Cisplatin (μM)	0	0.2	0.6	2.5	10
0	1	0.950	0.856	0.519	0.218
0.08	0.987	0.939	0.806	0.535	0.229
0.3	1.022	0.892	0.760	0.543	0.216
1.2	0.979	0.852	0.742	0.522	0.237
5	0.837	0.695	0.640	0.505	0.268

A673

Rucaparib (μM) \ Cisplatin (μM)	0	0.2	0.6	2.5	10
0	1	0.754	0.388	0.136	0.023
0.08	0.988	0.705	0.346	0.121	0.028
0.3	0.958	0.683	0.336	0.129	0.023
1.2	0.856	0.577	0.339	0.131	0.027
5	0.526	0.388	0.276	0.123	0.033

DU-145

Rucaparib (μM) \ Cisplatin (μM)	0	0.2	0.6	2.5	10
0	1	0.927	0.873	0.423	0.106
0.08	1.036	1.029	0.960	0.378	0.111
0.3	1.077	0.975	0.934	0.383	0.116
1.2	0.974	0.938	0.868	0.448	0.110
5	0.895	0.784	0.745	0.368	0.117

MHH-ES-1

Rucaparib (μM) \ Cisplatin (μM)	0	0.2	0.6	2.5	10
0	1	0.975	0.893	0.497	0.158
0.08	0.936	0.867	0.791	0.385	0.167
0.3	0.922	0.795	0.677	0.360	0.161
1.2	0.709	0.613	0.436	0.292	0.140
5	0.312	0.287	0.264	0.214	0.148

MDA-MB-436

Rucaparib (μM) \ Cisplatin (μM)	0	0.2	0.6	2.5	10
0	1	0.794	0.533	0.387	0.184
0.08	0.886	0.673	0.538	0.366	0.197
0.3	0.883	0.681	0.500	0.372	0.181
1.2	0.869	0.766	0.522	0.397	0.178
5	0.727	0.593	0.488	0.368	0.228

ES8

Rucaparib (μM) \ Cisplatin (μM)	0	0.2	0.6	2.5	10
0	1	0.990	0.737	0.055	0.017
0.08	0.979	0.896	0.551	0.045	0.020
0.3	0.973	0.764	0.420	0.037	0.017
1.2	0.790	0.574	0.234	0.036	0.019
5	0.129	0.070	0.049	0.024	0.021

OLAR5

Rucaparib (μM) \ Cisplatin (μM)	0	0.2	0.6	2.5	10
0	1	1.014	0.988	0.363	0.085
0.08	1.044	1.001	0.991	0.324	0.089
0.3	1.032	1.020	0.930	0.343	0.093
1.2	1.046	0.977	0.948	0.359	0.092
5	1.040	0.980	0.959	0.376	0.128

Rucaparib/Temozolomide

ES7 — Temozolomide (µM)

Rucaparib (µM)	0	3	12.5	50	200
0	1	0.847	0.889	0.855	0.363
0.08	0.942	0.860	0.722	0.325	0.035
0.3	0.841	0.828	0.610	0.161	0.023
1.2	0.688	0.571	0.317	0.068	0.023
5	0.163	0.137	0.051	0.030	0.021

U-2-OS — Temozolomide (µM)

Rucaparib (µM)	0	3	12.5	50	200
0	1	1.047	0.894	0.868	0.576
0.08	0.987	0.943	0.899	0.777	0.401
0.3	1.022	0.912	0.917	0.658	0.378
1.2	0.979	0.939	0.799	0.479	0.355
5	0.837	0.779	0.649	0.424	0.364

A673 — Temozolomide (µM)

Rucaparib (µM)	0	3	12.5	50	200
0	1	1.001	0.985	0.958	0.589
0.08	0.988	0.955	0.919	0.529	0.160
0.3	0.958	0.906	0.753	0.397	0.133
1.2	0.856	0.848	0.608	0.272	0.102
5	0.526	0.516	0.326	0.162	0.079

DU-145 — Temozolomide (µM)

Rucaparib (µM)	0	3	12.5	50	200
0	1	0.960	0.968	0.978	0.862
0.08	1.036	1.000	0.985	0.983	0.419
0.3	1.077	0.928	0.930	0.814	0.265
1.2	0.974	0.917	0.830	0.703	0.176
5	0.895	0.842	0.765	0.404	0.136

MHH-ES-1 — Temozolomide (µM)

Rucaparib (µM)	0	3	12.5	50	200
0	1	0.913	0.893	0.943	0.562
0.08	0.936	0.815	0.807	0.513	0.114
0.3	0.922	0.772	0.735	0.325	0.082
1.2	0.709	0.658	0.434	0.162	0.054
5	0.312	0.252	0.180	0.079	0.041

MDA-MB-436 — Temozolomide (µM)

Rucaparib (µM)	0	3	12.5	50	200
0	1	1.001	0.946	0.933	0.714
0.08	0.886	0.933	0.878	0.694	0.407
0.3	0.883	0.892	0.738	0.593	0.382
1.2	0.869	0.752	0.670	0.527	0.380
5	0.727	0.671	0.522	0.432	0.347

ES8 — Temozolomide (µM)

Rucaparib (µM)	0	3	12.5	50	200
0	1	0.986	0.985	0.933	0.522
0.08	0.979	0.851	0.896	0.394	0.018
0.3	0.973	0.912	0.791	0.130	0.017
1.2	0.790	0.827	0.443	0.028	0.015
5	0.129	0.114	0.029	0.025	0.016

OLAR5 — Temozolomide (µM)

Rucaparib (µM)	0	3	12.5	50	200
0	1	0.984	0.954	0.938	0.426
0.08	1.044	0.959	0.918	0.971	0.531
0.3	1.032	0.947	0.987	0.896	0.454
1.2	1.046	0.962	0.919	0.814	0.241
5	1.040	0.999	0.963	0.677	0.115

Rucaparib/MMS

ES7 — MMS (µM)

Rucaparib (µM)	0	8	31	125	500
0	1	0.940	0.858	0.504	0.020
0.08	0.866	0.261	0.034	0.014	0.012
0.3	0.741	0.113	0.022	0.012	0.012
1.2	0.536	0.039	0.014	0.011	0.011
5	0.127	0.020	0.015	0.013	0.013

U-2-OS — MMS (µM)

Rucaparib (µM)	0	8	31	125	500
0	1	0.977	0.972	0.788	0.345
0.08	1.020	0.920	0.683	0.412	0.259
0.3	0.985	0.845	0.488	0.412	0.248
1.2	0.962	0.659	0.441	0.389	0.254
5	0.786	0.492	0.429	0.398	0.239

A673 — MMS (µM)

Rucaparib (µM)	0	8	31	125	500
0	1	1.059	0.951	0.527	0.096
0.08	0.889	0.440	0.181	0.085	0.045
0.3	0.817	0.339	0.136	0.083	0.043
1.2	0.737	0.259	0.115	0.057	0.042
5	0.455	0.150	0.107	0.052	0.048

DU-145 — MMS (µM)

Rucaparib (µM)	0	8	31	125	500
0	1	0.975	1.010	0.951	0.334
0.08	0.968	0.973	0.650	0.155	0.080
0.3	0.971	0.895	0.390	0.147	0.068
1.2	0.939	0.783	0.236	0.132	0.068
5	0.863	0.421	0.164	0.102	0.057

MHH-ES-1 — MMS (µM)

Rucaparib (µM)	0	8	31	125	500
0	1	0.178	0.049	0.027	0.020
0.08	0.936	0.124	0.041	0.021	0.019
0.3	0.922	0.112	0.045	0.023	0.017
1.2	0.709	0.094	0.038	0.024	0.016
5	0.312	0.084	0.043	0.026	0.021

MDA-MB-436 — MMS (µM)

Rucaparib (µM)	0	8	31	125	500
0	1	0.978	0.951	0.748	0.180
0.08	0.985	0.631	0.582	0.336	0.158
0.3	0.812	0.580	0.417	0.248	0.159
1.2	0.790	0.513	0.390	0.254	0.132
5	0.671	0.426	0.365	0.233	0.132

ES8 — MMS (µM)

Rucaparib (µM)	0	8	31	125	500
0	1	1.008	0.979	0.626	0.007
0.08	0.918	0.189	0.012	0.006	0.005
0.3	0.844	0.073	0.007	0.005	0.005
1.2	0.597	0.012	0.006	0.005	0.005
5	0.083	0.008	0.006	0.005	0.005

OLAR5 — MMS (µM)

Rucaparib (µM)	0	8	31	125	500
0	1	1.034	0.891	0.129	0.024
0.08	1.089	0.989	0.818	0.083	0.023
0.3	1.067	1.014	0.610	0.052	0.025
1.2	1.071	0.972	0.299	0.045	0.020
5	0.986	0.617	0.107	0.040	0.020

BMN-673/Camptothecin

ES7 — Camptothecin (µM)

BMN-673 (µM)	0	0.002	0.006	0.03	0.1
0	1	0.107	0.028	0.024	0.020
0.002	0.874	0.038	0.026	0.022	0.022
0.006	0.434	0.030	0.025	0.023	0.018
0.02	0.165	0.027	0.023	0.021	0.017
0.1	0.071	0.026	0.021	0.020	0.020

U-2-OS — Camptothecin (µM)

BMN-673 (µM)	0	0.002	0.006	0.03	0.1
0	1	0.900	0.500	0.400	0.400
0.002	1.000	0.700	0.500	0.400	0.400
0.006	1.000	0.600	0.400	0.400	0.400
0.02	0.900	0.500	0.400	0.400	0.400
0.1	0.800	0.500	0.400	0.400	0.300

A673 — Camptothecin (µM)

BMN-673 (µM)	0	0.002	0.006	0.03	0.1
0	1	0.300	0.128	0.059	0.041
0.002	0.956	0.227	0.112	0.065	0.041
0.006	0.709	0.175	0.097	0.053	0.044
0.02	0.485	0.152	0.085	0.049	0.037
0.1	0.332	0.126	0.068	0.062	0.038

DU-145 — Camptothecin (µM)

BMN-673 (µM)	0	0.002	0.006	0.03	0.1
0	1	0.949	0.329	0.152	0.117
0.002	1.062	0.951	0.246	0.142	0.109
0.006	1.013	0.677	0.205	0.138	0.115
0.02	0.947	0.493	0.190	0.130	0.109
0.1	0.852	0.335	0.157	0.140	0.101

MHH-ES-1 — Camptothecin (µM)

BMN-673 (µM)	0	0.002	0.006	0.03	0.1
0	1	0.251	0.071	0.031	0.024
0.002	0.946	0.167	0.053	0.030	0.019
0.006	0.661	0.116	0.056	0.030	0.026
0.02	0.345	0.094	0.049	0.026	0.028
0.1	0.230	0.075	0.050	0.030	0.025

MDA-MB-436 — Camptothecin (µM)

BMN-673 (µM)	0	0.002	0.006	0.03	0.1
0	1	0.797	0.468	0.384	0.316
0.002	0.893	0.600	0.464	0.331	0.286
0.006	0.737	0.560	0.395	0.320	0.277
0.02	0.641	0.534	0.426	0.313	0.276
0.1	0.590	0.418	0.395	0.326	0.275

ES8 — Camptothecin (µM)

BMN-673 (µM)	0	0.002	0.006	0.03	0.1
0	1	0.163	0.021	0.017	0.015
0.002	0.935	0.022	0.017	0.015	0.016
0.006	0.649	0.022	0.015	0.015	0.012
0.02	0.186	0.019	0.020	0.012	0.012
0.1	0.035	0.019	0.014	0.011	0.018

OLAR5 — Camptothecin (µM)

BMN-673 (µM)	0	0.002	0.006	0.03	0.1
0	1	0.134	0.050	0.039	0.031
0.002	1.004	0.127	0.056	0.036	0.035
0.006	1.025	0.110	0.053	0.036	0.037
0.02	1.035	0.120	0.056	0.034	0.032
0.1	1.025	0.123	0.057	0.037	0.038

BMN-673/Cisplatin

ES7 — Cisplatin (µM)

BMN-673 (µM)	0	0.2	0.6	2.5	10
0	1	0.877	0.503	0.101	0.039
0.002	0.874	0.675	0.327	0.099	0.037
0.006	0.434	0.283	0.166	0.069	0.040
0.02	0.165	0.110	0.081	0.051	0.034
0.1	0.071	0.059	0.059	0.048	0.029

U-2-OS — Cisplatin (µM)

BMN-673 (µM)	0	0.2	0.6	2.5	10
0	1	0.976	0.877	0.604	0.271
0.002	1.017	0.941	0.809	0.559	0.275
0.006	1.000	0.907	0.730	0.569	0.277
0.02	0.910	0.754	0.668	0.550	0.279
0.1	0.760	0.644	0.554	0.473	0.261

A673 — Cisplatin (µM)

BMN-673 (µM)	0	0.2	0.6	2.5	10
0	1	0.852	0.457	0.150	0.030
0.002	0.956	0.653	0.379	0.154	0.029
0.006	0.709	0.507	0.328	0.132	0.029
0.02	0.485	0.355	0.253	0.136	0.029
0.1	0.332	0.296	0.234	0.118	0.029

DU-145 — Cisplatin (µM)

BMN-673 (µM)	0	0.2	0.6	2.5	10
0	1	0.966	0.833	0.478	0.123
0.002	1.062	1.008	0.905	0.474	0.134
0.006	1.013	0.980	0.874	0.430	0.136
0.02	0.947	0.825	0.802	0.364	0.112
0.1	0.852	0.773	0.657	0.307	0.125

MHH-ES-1 — Cisplatin (µM)

BMN-673 (µM)	0	0.2	0.6	2.5	10
0	1	0.953	0.943	0.533	0.178
0.002	0.946	0.907	0.738	0.420	0.166
0.006	0.661	0.580	0.471	0.293	0.163
0.02	0.345	0.328	0.290	0.235	0.147
0.1	0.230	0.194	0.202	0.179	0.112

MDA-MB-436 — Cisplatin (µM)

BMN-673 (µM)	0	0.2	0.6	2.5	10
0	1	0.749	0.586	0.431	0.207
0.002	0.893	0.683	0.535	0.513	0.205
0.006	0.737	0.587	0.497	0.447	0.223
0.02	0.641	0.628	0.477	0.404	0.215
0.1	0.590	0.555	0.499	0.409	0.203

ES8 — Cisplatin (µM)

BMN-673 (µM)	0	0.2	0.6	2.5	10
0	1	0.990	0.744	0.073	0.018
0.002	0.935	0.844	0.487	0.057	0.020
0.006	0.649	0.383	0.149	0.030	0.019
0.02	0.186	0.081	0.041	0.025	0.017
0.1	0.035	0.031	0.030	0.022	0.017

OLAR5 — Cisplatin (µM)

BMN-673 (µM)	0	0.2	0.6	2.5	10
0	1	1.002	0.883	0.386	0.098
0.002	1.004	0.987	1.014	0.382	0.098
0.006	1.025	0.939	0.956	0.362	0.098
0.02	1.035	1.030	0.961	0.362	0.091
0.1	1.025	0.938	0.897	0.329	0.086

BMN-673/Temozolomide

ES7 — Temozolomide (µM)

BMN-673 (µM)	0	3	12.5	50	200
0	1	0.947	0.951	0.831	0.598
0.002	0.874	0.815	0.616	0.175	0.034
0.006	0.434	0.393	0.193	0.039	0.022
0.02	0.165	0.143	0.060	0.025	0.023
0.1	0.071	0.062	0.032	0.026	0.021

U-2-OS — Temozolomide (µM)

BMN-673 (µM)	0	3	12.5	50	200
0	1	1.017	0.981	0.860	0.615
0.002	1.017	0.985	0.949	0.825	0.396
0.006	1.000	0.889	0.759	0.477	0.385
0.02	0.910	0.836	0.670	0.403	0.338
0.1	0.760	0.742	0.536	0.423	0.364

A673 — Temozolomide (µM)

BMN-673 (µM)	0	3	12.5	50	200
0	1	1.010	1.016	1.034	0.773
0.002	0.956	0.971	0.803	0.497	0.175
0.006	0.709	0.734	0.522	0.202	0.097
0.02	0.485	0.489	0.287	0.154	0.072
0.1	0.332	0.292	0.218	0.122	0.069

DU-145 — Temozolomide (µM)

BMN-673 (µM)	0	3	12.5	50	200
0	1	0.911	1.027	0.978	0.931
0.002	1.062	0.943	0.932	0.879	0.525
0.006	1.013	0.983	0.925	0.587	0.172
0.02	0.947	0.870	0.769	0.312	0.152
0.1	0.852	0.765	0.544	0.187	0.140

MHH-ES-1 — Temozolomide (µM)

BMN-673 (µM)	0	3	12.5	50	200
0	1	0.904	0.896	0.918	0.772
0.002	0.946	0.844	0.775	0.485	0.127
0.006	0.661	0.624	0.463	0.138	0.052
0.02	0.345	0.331	0.206	0.088	0.042
0.1	0.230	0.215	0.134	0.070	0.034

MDA-MB-436 — Temozolomide (µM)

BMN-673 (µM)	0	3	12.5	50	200
0	1	0.958	1.022	0.943	0.814
0.002	0.893	0.895	0.811	0.590	0.424
0.006	0.737	0.677	0.652	0.451	0.330
0.02	0.641	0.604	0.536	0.425	0.332
0.1	0.590	0.550	0.489	0.377	0.324

ES8 — Temozolomide (µM)

BMN-673 (µM)	0	3	12.5	50	200
0	1	0.975	1.012	0.925	0.773
0.002	0.935	0.800	0.763	0.224	0.018
0.006	0.649	0.595	0.238	0.023	0.015
0.02	0.186	0.158	0.026	0.018	0.018
0.1	0.035	0.029	0.021	0.016	0.016

OLAR5 — Temozolomide (µM)

BMN-673 (µM)	0	3	12.5	50	200
0	1	0.978	0.916	0.984	0.620
0.002	1.004	1.018	1.047	0.977	0.531
0.006	1.025	0.940	0.972	0.899	0.456
0.02	1.035	0.945	0.868	0.892	0.260
0.1	1.025	0.996	0.915	0.863	0.193

BMN-673/MMS

ES7 — MMS (µM)

BMN-673 (µM)	0	8	31	125	500
0	1	0.985	0.918	0.430	0.019
0.002	0.715	0.054	0.021	0.015	0.012
0.006	0.212	0.017	0.014	0.013	0.013
0.02	0.067	0.016	0.014	0.012	0.012
0.1	0.032	0.014	0.013	0.013	0.013

U-2-OS — MMS (µM)

BMN-673 (µM)	0	8	31	125	500
0	1	0.982	0.967	0.871	0.312
0.002	1.017	0.879	0.517	0.437	0.261
0.006	0.950	0.506	0.441	0.399	0.223
0.02	0.804	0.477	0.471	0.358	0.201
0.1	0.664	0.487	0.419	0.329	0.208

A673 — MMS (µM)

BMN-673 (µM)	0	8	31	125	500
0	1	0.969	0.963	0.503	0.082
0.002	0.780	0.269	0.159	0.076	0.045
0.006	0.482	0.132	0.091	0.048	0.046
0.02	0.341	0.126	0.075	0.044	0.038
0.1	0.236	0.101	0.060	0.041	0.035

DU-145 — MMS (µM)

BMN-673 (µM)	0	8	31	125	500
0	1	1.044	1.054	0.964	0.346
0.002	1.016	0.799	0.360	0.154	0.071
0.006	0.924	0.253	0.162	0.111	0.073
0.02	0.781	0.199	0.140	0.101	0.064
0.1	0.661	0.166	0.123	0.101	0.060

MHH-ES-1 — MMS (µM)

BMN-673 (µM)	0	8	31	125	500
0	1	1.032	0.923	0.637	0.142
0.002	0.879	0.245	0.120	0.042	0.045
0.006	0.443	0.077	0.043	0.030	0.043
0.02	0.218	0.056	0.034	0.028	0.042
0.1	0.146	0.039	0.027	0.026	0.036

MDA-MB-436 — MMS (µM)

BMN-673 (µM)	0	8	31	125	500
0	1	1.022	0.980	0.711	0.223
0.002	0.893	0.680	0.432	0.290	0.160
0.006	0.705	0.399	0.385	0.264	0.132
0.02	0.610	0.392	0.353	0.246	0.128
0.1	0.538	0.381	0.285	0.240	0.129

ES8 — MMS (µM)

BMN-673 (µM)	0	8	31	125	500
0	1	0.946	0.992	0.507	0.007
0.002	0.850	0.031	0.007	0.005	0.004
0.006	0.361	0.007	0.005	0.005	0.004
0.02	0.049	0.006	0.006	0.005	0.003
0.1	0.012	0.005	0.004	0.004	0.003

OLAR5 — MMS (µM)

BMN-673 (µM)	0	8	31	125	500
0	1	0.974	0.874	0.128	0.039
0.002	1.045	0.936	0.668	0.091	0.041
0.006	1.005	0.868	0.540	0.078	0.032
0.02	1.053	0.906	0.364	0.055	0.031
0.1	0.990	0.817	0.217	0.050	0.031
