# Supplementary material for: Combinations of PARP Inhibitors with Temozolomide Drive PARP1 Trapping and Apoptosis in Ewing’s Sarcoma
Source: PLoS One. 2015 Oct 27;10(10):e0140988. doi: 10.1371/journal.pone.0140988 (PMC4624427; doi:10.1371/journal.pone.0140988)
Supplement: S1 Text — (DOCX) [file pone.0140988.s010.docx]

**Supplementary Methods**

*Cell lines*

RPE-1, U-2-OS, MDA-MB-436, DU-145, HelaSF, RPMI-7951, HRC575, NCI-H2342, CAPAN-1, A673, MHH-ES-1, MC-IXC, SK-ES-1, SK-NEP-1, SK-PN-DW, TC-71 and CADO-ES1 human cell lines were purchased from commercial vendors. ES1, ES3, ES4, ES5, ES6, ES7, ES8 and RH-1 were kindly donated by Susan Ragsdale and EW-1, EW-3, EW-7, EW-11, EW-13, EW-16, EW-18, EW-22 and EW-24 by Professor Gilbert Lenoir. Cell lines were cultured in DMEM/F12 (MDA-MB-436, DU-145, A673, RPE-1, NCI-H2342, CAPAN-1, MC-IXC, SK-PN-DW, TC-71 and HelaSF) or RPMI (ES7, ES8, MHH-ES-1, U-2-OS, HRC575, RPMI-7951, ES1, ES3, ES4, ES5, ES6, EW-1, EW-3, EW-7, EW-11, EW-13, EW-16, EW-18, EW-22, EW-24, RH-1, SK-ES-1, SK-NEP-1, CADO-ES-1) medium supplemented with 5% FBS (or 10% for HRC575 and RPE-1) and penicillin/streptomycin, and maintained at 37^o^C in a humidified atmosphere at 5% CO_2_. OLAR5 cells were grown as parental ES8 cells but in the presence of 5μM olaparib. Cell lines were propagated every 3-4 days. All cell lines, including OLAR5 cells, were SNP- and STR-profiled to exclude cross-contamination as previously described(7). Complete cell line information is available on COSMIC (http://cancer.sanger.ac.uk/cancergenome/projects/cosmic/).

*Western blotting and immunoprecipitation*

Cells were plated at a density of 1.5-3x10^5^ cells in 6-well plates in complete growth medium. Cells were treated with drug as indicated and lysates prepared in NP40 extraction buffer supplemented with phosphatase and protease inhibitor cocktails (Sigma and Cell Signaling) or a cellular sub-fractionation assay performed using a kit (Thermo Fisher Scientific) as per manufacturers instructions. The concentration of protein cell lysates was determined by protein assay using purified bovine serum albumin (BSA) as a standard according to manufacturer’s instructions (Bio-Rad Laboratories). Lysates were subjected to SDS-PAGE electrophoresis and western botted with the following antibodies: phospho-CHK2 (T68), anti-phospho-CHK1 (S345; 133D3), anti-γH2AX (S139) and anti-53BP1 from Cell Signaling, anti-phospho-ATM (S1981) from Epitomics, anti-phospho-KAP1 (S824) and anti-phospho-RPA (S4/8) from Bethyl Laboratories, anti-phospho-KAP1 (S473) from BioLegend, anti-H3 and anti-GAPDH from Millipore, anti-MRE11 and anti-RPA from Abcam and anti-PARP1, anti-CHK1, anti-CHK2, anti-ATR and anti-ATM from Santa Cruz Biotechnologies and anti-tubulin from Sigma. BRCA1/2 immunoprecipitation was performed from 300μl NP40 cell lysates from three 10cm Petri dishes. BRCA1 was immunoprecipitated using 5μg of anti-BRCA1 (C-20) antibody from Santa Cruz Biotechnologies and BRCA2 using 5μg of anti-BRCA2 (Ab-1) antibody from Calbiochem. The antibody-protein complex was captured using 20μl of a 1:1 mix of protein G sepharose 4B beads (Sigma) in PBS and immunoprecipitates mixed for 2h at 4^o^C on a rotation wheel. Thereafter immunoprecipitates were washed three times with 300μl NP40 lysis buffer before analysis by SDS-PAGE. Western blotting was performed for BRCA1 with an anti-BRCA1 antibody from Millipore and an anti-BRCA2 antibody from Calbiochem.

*Immunofluorescence*

Cell treatments and immunofluorescent staining for analysis on the Cellomics Arrayscan were performed as described in the main methods section. For analysis of 53BP1 foci formation, cells were stained with a 1:500 dilution of anti-53BP1 antibody (Cell Signaling) and cells scored as being positive if they had more than five 53BP1 foci per nucleus.

*Flow cytometry*

Cells were plated in 6-well plates 48h prior to drug treatment. Cells were co-pulsed with 5μM EdU and washed with PBS and overlaid with fresh media overnight before harvesting by trypsin. Harvested cells were fixed and permeabilized with 2% paraformaldehyde/0.2% Triton-X-100, blocked with 3% BSA/PBS, then subjected to Click-IT as per manufacturers instructions (LifeTechnologies). Cells were washed in between steps with PBS. Following Click-IT, cells were subject to *in situ* cell death detection (TMR red, Roche) for the end-labeling of apoptotic DNA DSBs (specificity determined by co-treatment with the caspase inhibitor Z-VAD, data not shown), as per manufacturers instructions. Cells were then washed and resuspended in PBS containing 2μg/ml DAPI (Sigma) and analyzed on a BD Fortessa. All subsequent flow cytometry analyses were performed on FlowJo, where samples were double-gated for doublets by FS-A/SS-A and DAPI/FS-W prior to apoptotic, DNA content and EdU incorporation analysis.

*Confirmation of disruption of the EWS gene in EWSCs*

Interphase and metaphase fluorescent in situ hybridization (FISH) was performed using the Vysis EWSR1 (22q12) Dual Color Break Apart Rearrangement Probe to detect the disruption of the *EWS* gene as described by the manufacturer (Abbott Molecular). In a subset of Ewing’s cell lines, disruption of *EWS* was detected using PCR or RNA sequencing as previously described(7).
